# Supplementary material for: Elevation-dependent intensification of fire danger in the western United States
Source: Nat Commun. 2023 Mar 30;14:1773. doi: 10.1038/s41467-023-37311-4 (PMC10063545; doi:10.1038/s41467-023-37311-4)
Supplement: Supplementary file 1 — Supplementary Information [file 41467_2023_37311_MOESM1_ESM.pdf]

Supplementary Information

For

Elevation-dependent intensification of fire danger in the western United States

Mohammad Reza Alizadeh[1,2], John T. Abatzoglou[3], Jan Adamowski[1], Arash Modaresi Rad[4], Amir AghaKouchak[5,6], Francesco S. R. Pausata[7], Mojtaba Sadegh[4]

[1] Department of Bioresource Engineering, McGill University, Quebec, CANADA.
[2] Department of Civil and Environmental Engineering, Massachusetts Institute of Technology, Cambridge, MA, UNITED STATES.
[3] Management of Complex Systems Department, University of California, Merced, Merced, CA, UNITED STATES.
[4] Department of Civil Engineering, Boise State University, Boise, ID, UNITED STATES
[5] Department of Civil and Environmental Engineering, University of California, Irvine, CA, UNITED STATES.
[6] Department of Earth System Science, University of California, Irvine, CA, UNITED STATES.
[7] Department of Earth and Atmospheric Sciences, University of Quebec in Montreal, Montreal, QC, CANADA

Table S1. Increase in annual critical fire danger days (ERC≥60) from 1979-2020 in each elevation band in each ecoregion.

	0-500 m	500-1000 m	1000-1500 m	1500-2000 m	2000-2500 m	2500-3000 m	>3000 m
4: Cascades	3	6	14	25	34	NAN	NAN
5: Sierra Nevada	64	64	59	62	71	81	88
11: Blue Mountains	39	34	35	36	33	NAN	NAN
13: Central Basin and Range	NAN	NAN	39	60	74	87	97
15: Northern Rockies	21	23	24	25	23	NAN	NAN
16: Idaho Batholith	NAN	35	30	30	29	28	29
17: Middle Rockies	NAN	25	14	19	20	21	22
19: Wasatch and Unita Mountains	NAN	NAN	NAN	45	54	58	47
20: Colorado Plateaus	NAN	NAN	58	65	66	63	NAN
21: Southern Rockies	NAN	NAN	NAN	67	66	59	48
22: Arizona/New Mexico Plateau	NAN	58	71	79	62	70	NAN
23: Arizona/New Mexico Mountains	NAN	104	100	93	82	88	78
41: Canadian Rockies	NAN	NAN	13	12	12	NAN	NAN
77: North Cascades	2	9	7	6	7	NAN	NAN
78: Klamath Mountains	24	30	36	44	50	NAN	NAN

Table S2. Increase in warm-season (May-September) critical fire danger days (ERC$\geq$60) from 1979-2020 in each elevation band in each ecoregion.

	0-500 m	500-1000 m	1000-1500 m	1500-2000 m	2000-2500 m	2500-3000 m	>3000 m
4: Cascades	3	6	12	21	29	NAN	NAN
5: Sierra Nevada	34	35	29	36	43	48	53
11: Blue Mountains	39	34	35	36	33	NAN	NAN
13: Central Basin and Range	NAN	NAN	25	36	44	53	59
15: Northern Rockies	21	23	24	25	23	NAN	NAN
16: Idaho Batholith	NAN	35	30	30	29	28	29
17: Middle Rockies	NAN	24	13	19	20	21	21
19: Wasatch and Unita Mountains	NAN	NAN	NAN	39	43	45	38
20: Colorado Plateaus	NAN	NAN	17	29	39	41	NAN
21: Southern Rockies	NAN	NAN	NAN	45	43	41	36
22: Arizona/New Mexico Plateau	NAN	11	19	29	31	45	NAN
23: Arizona/New Mexico Mountains	NAN	31	30	29	29	31	33
41: Canadian Rockies	NAN	NAN	13	12	12	NAN	NAN
77: North Cascades	2	9	7	6	7	NAN	NAN
78: Klamath Mountains	21	23	25	30	37	NAN	NAN

Table S3. Increase in critical fire danger days (ERC≥60) outside May-September from 1979-2020 in each elevation band in each ecoregion.

	0-500 m	500-1000 m	1000-1500 m	1500-2000 m	2000-2500 m	2500-3000 m	>3000 m
4: Cascades	0	0	2	4	5	NAN	NAN
5: Sierra Nevada	30	29	30	26	28	33	35
11: Blue Mountains	0	0	0	0	0	NAN	NAN
13: Central Basin and Range	NAN	NAN	14	24	30	34	38
15: Northern Rockies	0	0	0	0	0	NAN	NAN
16: Idaho Batholith	NAN	0	0	0	0	0	0
17: Middle Rockies	NAN	1	1	0	0	0	1
19: Wasatch and Unita Mountains	NAN	NAN	NAN	6	11	13	9
20: Colorado Plateaus	NAN	NAN	41	36	27	22	NAN
21: Southern Rockies	NAN	NAN	NAN	22	23	18	12
22: Arizona/New Mexico Plateau	NAN	47	52	50	31	25	NAN
23: Arizona/New Mexico Mountains	NAN	73	70	64	53	57	46
41: Canadian Rockies	NAN	NAN	0	0	0	NAN	NAN
77: North Cascades	0	0	0	0	0	NAN	NAN
78: Klamath Mountains	3	7	11	14	13	NAN	NAN

Table S4. Land surface area (km^2) encapsulated in each elevation band in each ecoregion.

	0-500 m	500-1000 m	1000-1500 m	1500-2000 m	2000-2500 m	2500-3000 m	>3000 m
4: Cascades	7,025	17,145	19,437	13,074	1,963	143	66
5: Sierra Nevada	320	5,867	10,536	14,094	10,632	6,842	4,812
11: Blue Mountains	543	12,534	38,748	16,857	2,054	174	0
13: Central Basin and Range	0	29	99,301	149,569	50,607	8,016	1,267
15: Northern Rockies	1,078	33,126	34,921	12,331	503	1	0
16: Idaho Batholith	25	1,320	9,455	22,146	21,897	5,104	335
17: Middle Rockies	0	469	23,709	48,052	53,384	30,459	8,387
19: Wasatch and Unita Mountains	0	0	190	7,210	18,518	14,644	5,130
20: Colorado Plateaus	0	15	24,004	72,597	35,980	3,952	26
21: Southern Rockies	0	0	16	6,712	48,048	54,798	36,126
22: Arizona/New Mexico Plateau	0	368	9,982	90,060	45,204	1,243	1
23: Arizona/New Mexico Mountains	0	1,192	16,380	39,758	44,376	8,922	278
41: Canadian Rockies	0	122	4,344	9,620	4,617	175	1
77: North Cascades	3,135	8,140	9,931	7,295	1,843	33	2
78: Klamath Mountains	8,961	20,517	13,460	4,759	652	8	0

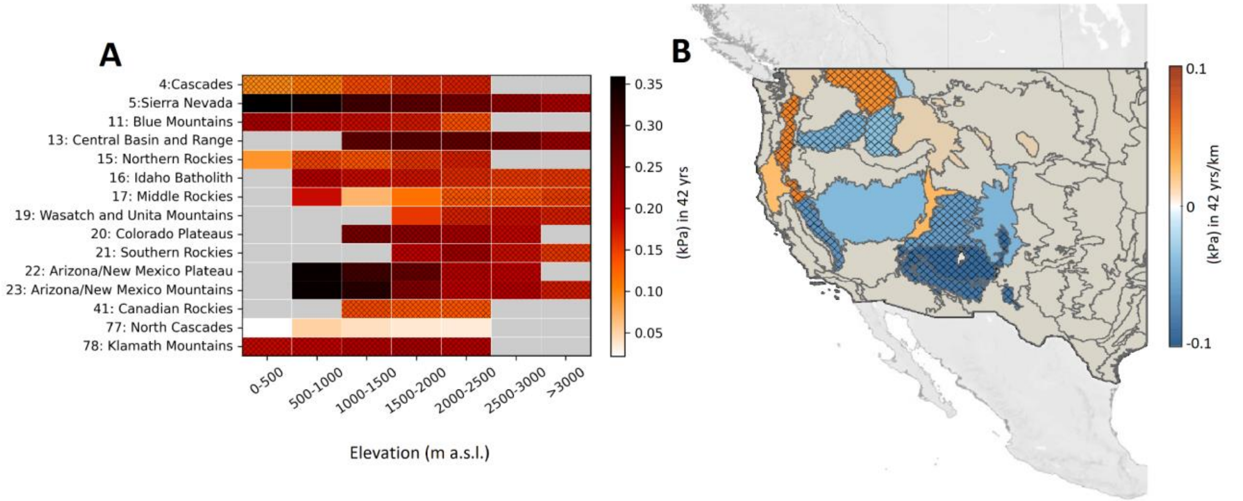

Figure S1. Elevation-dependent intensification of vapor pressure deficit, VPD. (A) *Temporal trends in warm-season average* ***VPD*** *from 1979-2020 in each elevation band in each mountainous ecoregion of the western US.* ***(B)*** *Slope of temporal* ***VPD*** *trends across elevation bands. (© OpenStreetMap contributors 2017. Distributed under the Open Data Commons Open Database License (ODbL) v1.0.)*[35]*.*

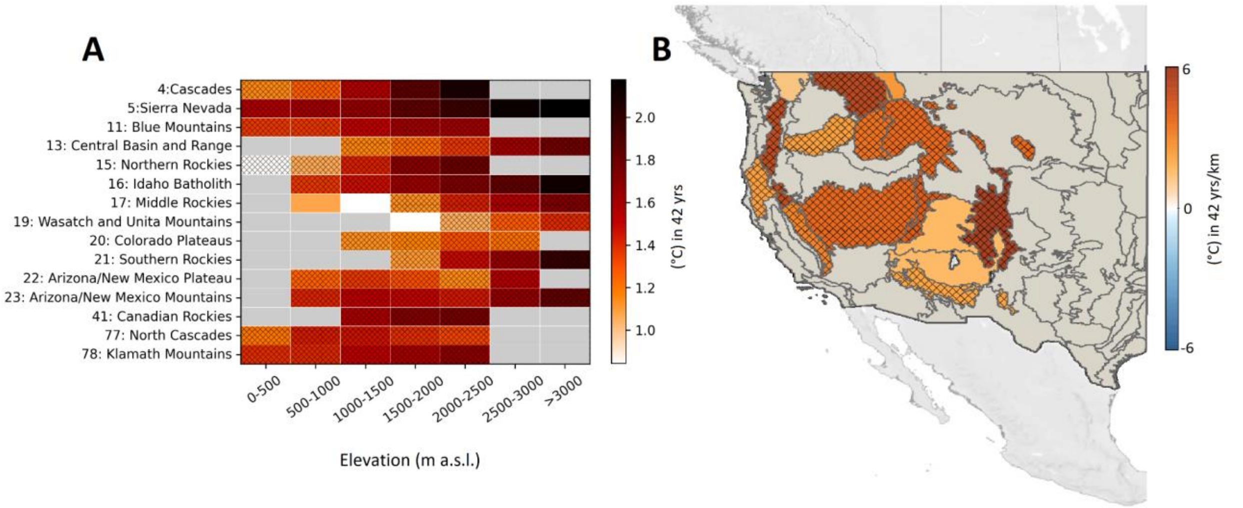

Figure S2. Elevation-dependent intensification of Temperature. (A) *Temporal trends in warm-season average daily* ***Temperature*** *from 1979-2020 in each elevation band in each mountainous ecoregion of the western US.* ***(B)*** *Slope of temporal* ***Temperature*** *trends across elevation bands. (© OpenStreetMap contributors 2017. Distributed under the Open Data Commons Open Database License (ODbL) v1.0.)*[35]*.*

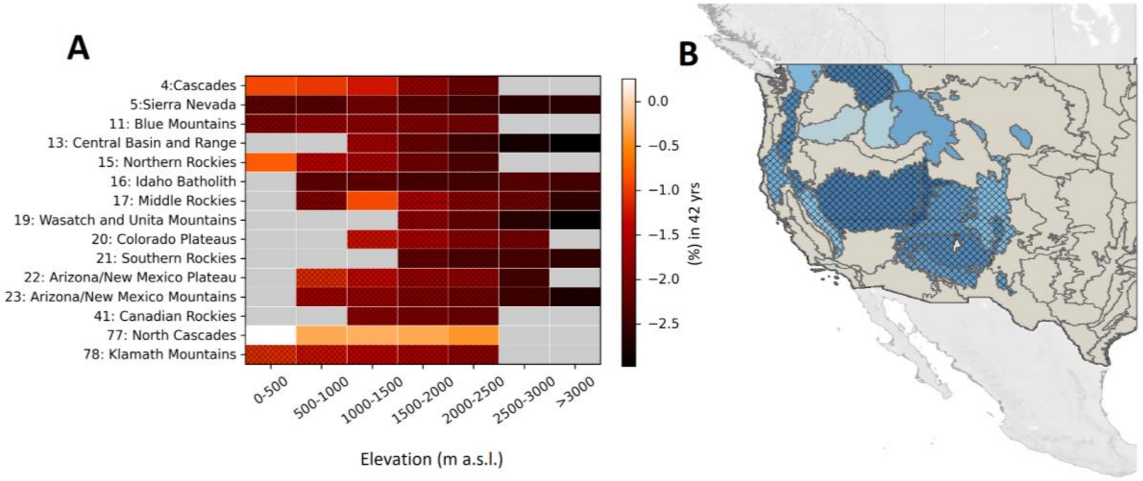

Figure S3. Elevation-dependent intensification of 100-hour dead fuel moisture, FM100. (A) *Temporal trends in warm-season **FM100** from 1979-2020 in each elevation band in each mountainous ecoregion of the western US. **(B)** Slope of temporal **FM100** trends across elevation bands. (© OpenStreetMap contributors 2017. Distributed under the Open Data Commons Open Database License (ODbL) v1.0.)*[35].

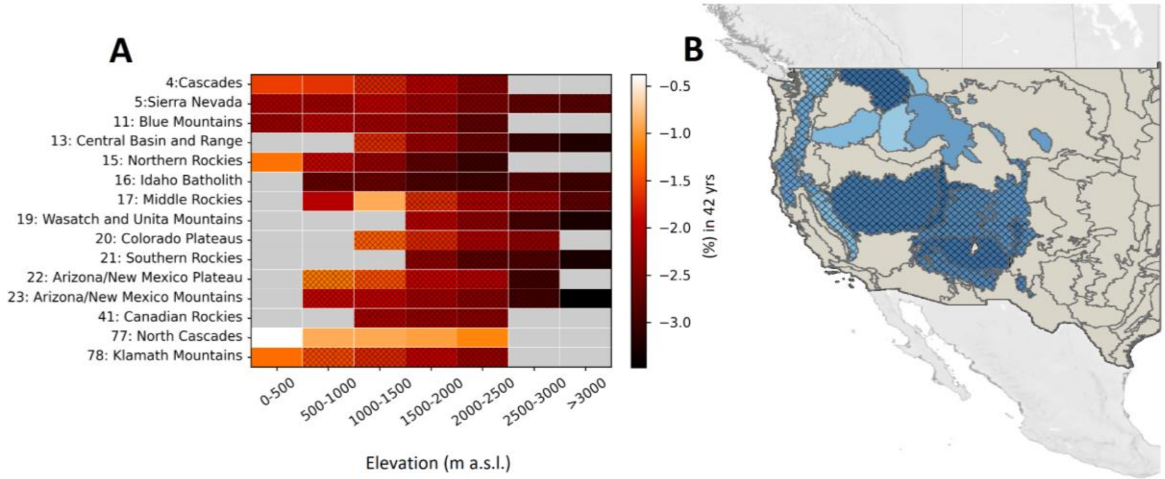

Figure S4. Elevation-dependent intensification of 1000-hour dead fuel moisture, FM1000. (A) *Temporal trends in warm-season **FM1000** from 1979-2020 in each elevation band in each mountainous ecoregion of the western US. **(B)** Slope of temporal **FM1000** trends across elevation bands. (© OpenStreetMap contributors 2017. Distributed under the Open Data Commons Open Database License (ODbL) v1.0.)*[35].

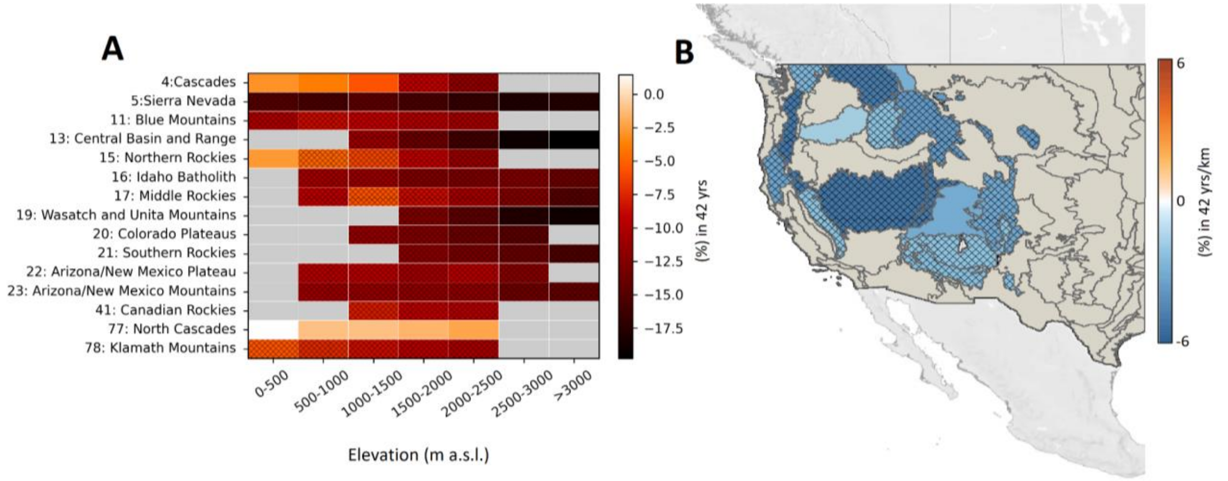

Figure S5. Elevation-dependent intensification of warm-season daily maximum relative humidity. (A) *Temporal trends in warm-season* ***daily maximum relative humidity*** *from 1979-2020 in each elevation band in each mountainous ecoregion of the western US.* ***(B)*** *Slope of temporal* ***daily maximum relative humidity*** *trends across elevation bands. (© OpenStreetMap contributors 2017. Distributed under the Open Data Commons Open Database License (ODbL) v1.0.)*[35]*.*

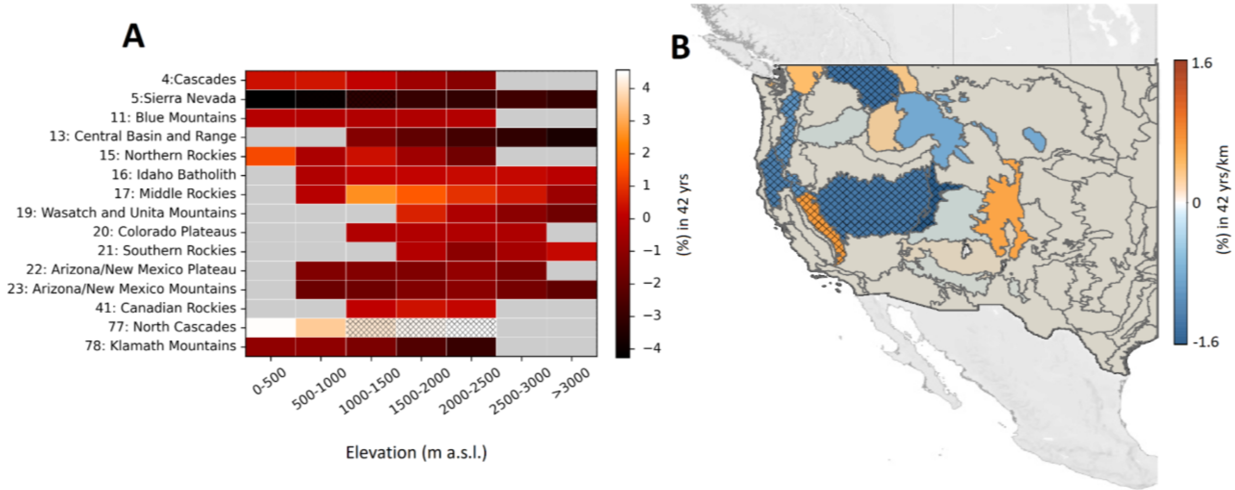

Figure S6. Elevation-dependent intensification of warm-season daily minimum relative humidity. (A) *Temporal trends in warm-season* ***daily minimum relative humidity*** *from 1979-2020 in each elevation band in each mountainous ecoregion of the western US.* ***(B)*** *Slope of temporal* ***daily minimum relative humidity*** *trends across elevation bands. (© OpenStreetMap contributors 2017. Distributed under the Open Data Commons Open Database License (ODbL) v1.0.)*[35]*.*

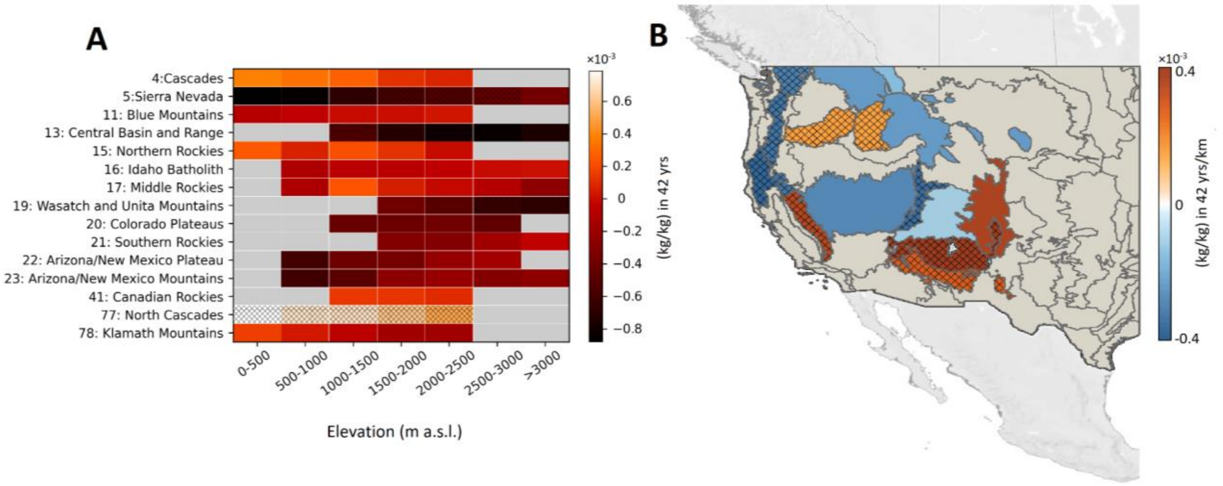

Figure S7. Elevation-dependent intensification of warm-season daily specific humidity. (A) *Temporal trends in warm-season* ***daily specific humidity*** *from 1979-2020 in each elevation band in each mountainous ecoregion of the western US.* ***(B)*** *Slope of temporal* ***daily specific humidity*** *trends across elevation bands. (© OpenStreetMap contributors 2017. Distributed under the Open Data Commons Open Database License (ODbL) v1.0.)*[35].

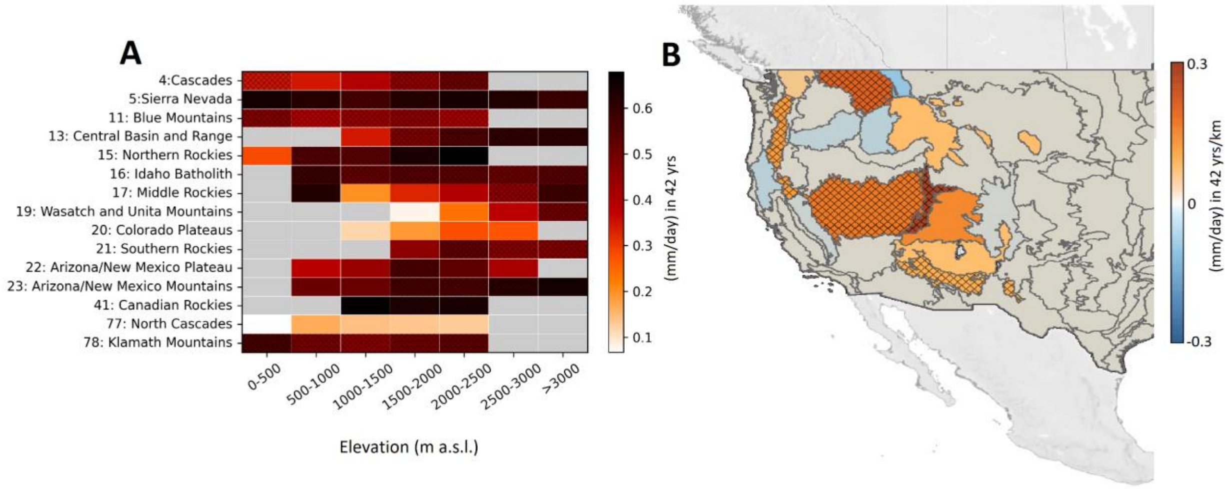

Figure S8. Elevation-dependent intensification of warm-season daily reference evapotranspiration. (A) *Temporal trends in warm-season* ***daily reference evapotranspiration*** *from 1979-2020 in each elevation band in each mountainous ecoregion of the western US.* ***(B)*** *Slope of temporal* ***daily reference evapotranspiration*** *trends across elevation bands. (© OpenStreetMap contributors 2017. Distributed under the Open Data Commons Open Database License (ODbL) v1.0.)*[35].

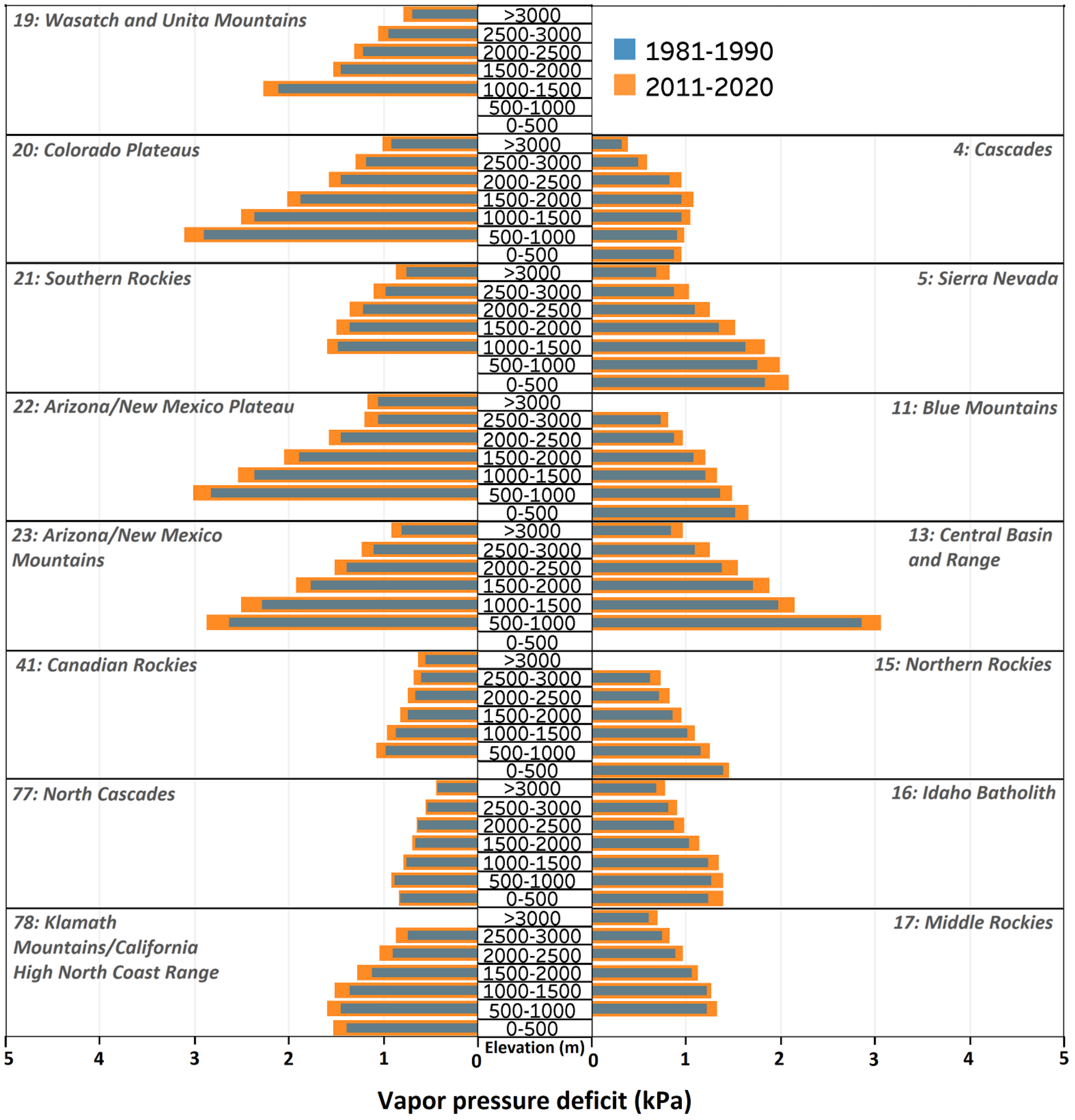

Figure S9. Decadal average warm-season VPD in each elevation band in each ecoregion*. Results for 1981-1990 and 2011-2020 are shown in blue and orange colors, respectively.*

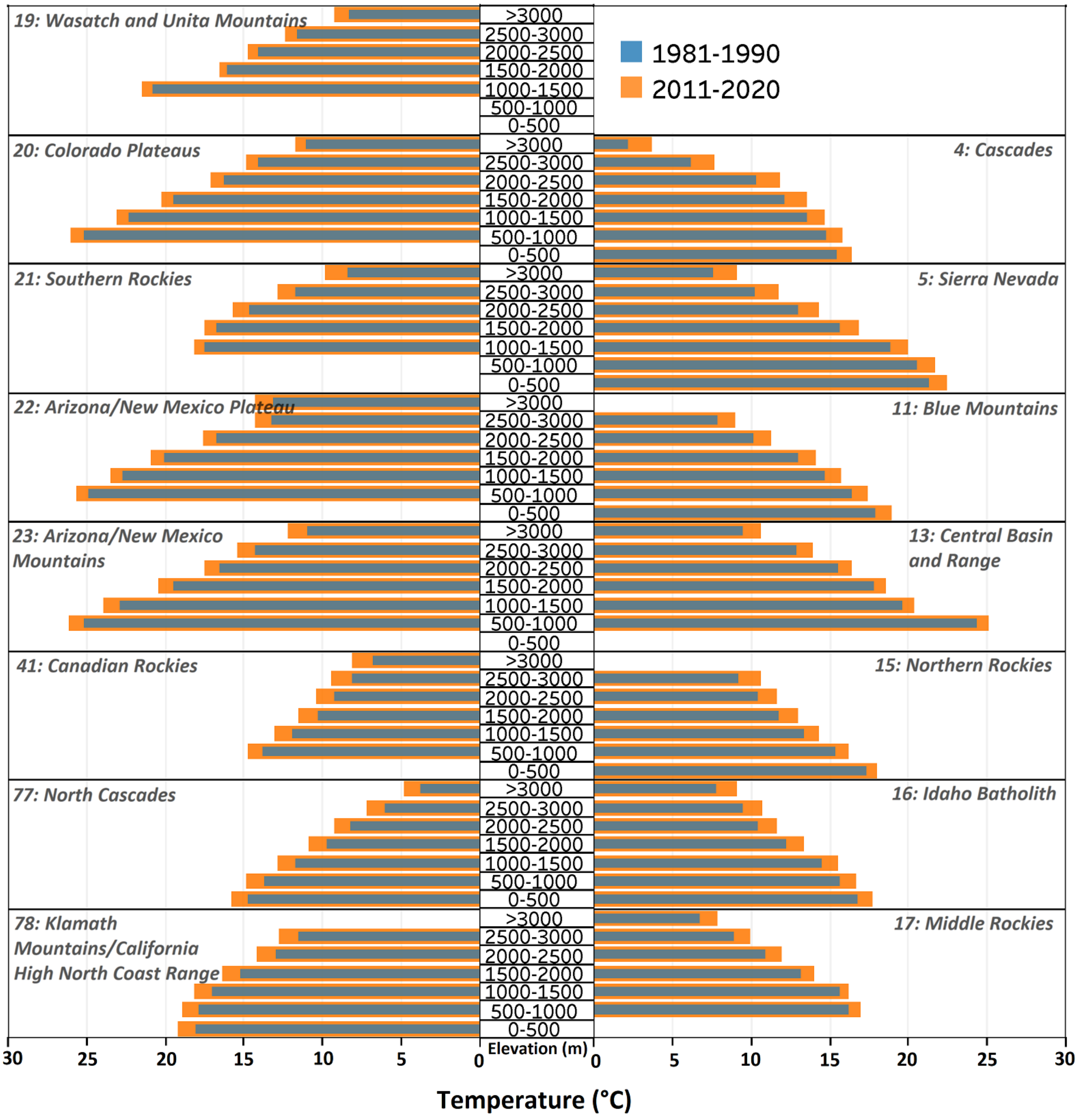

Figure S10. Decadal average warm-season Temperature in each elevation band in each ecoregion. *Results for 1981-1990 and 2011-2020 are shown in blue and orange colors, respectively.*

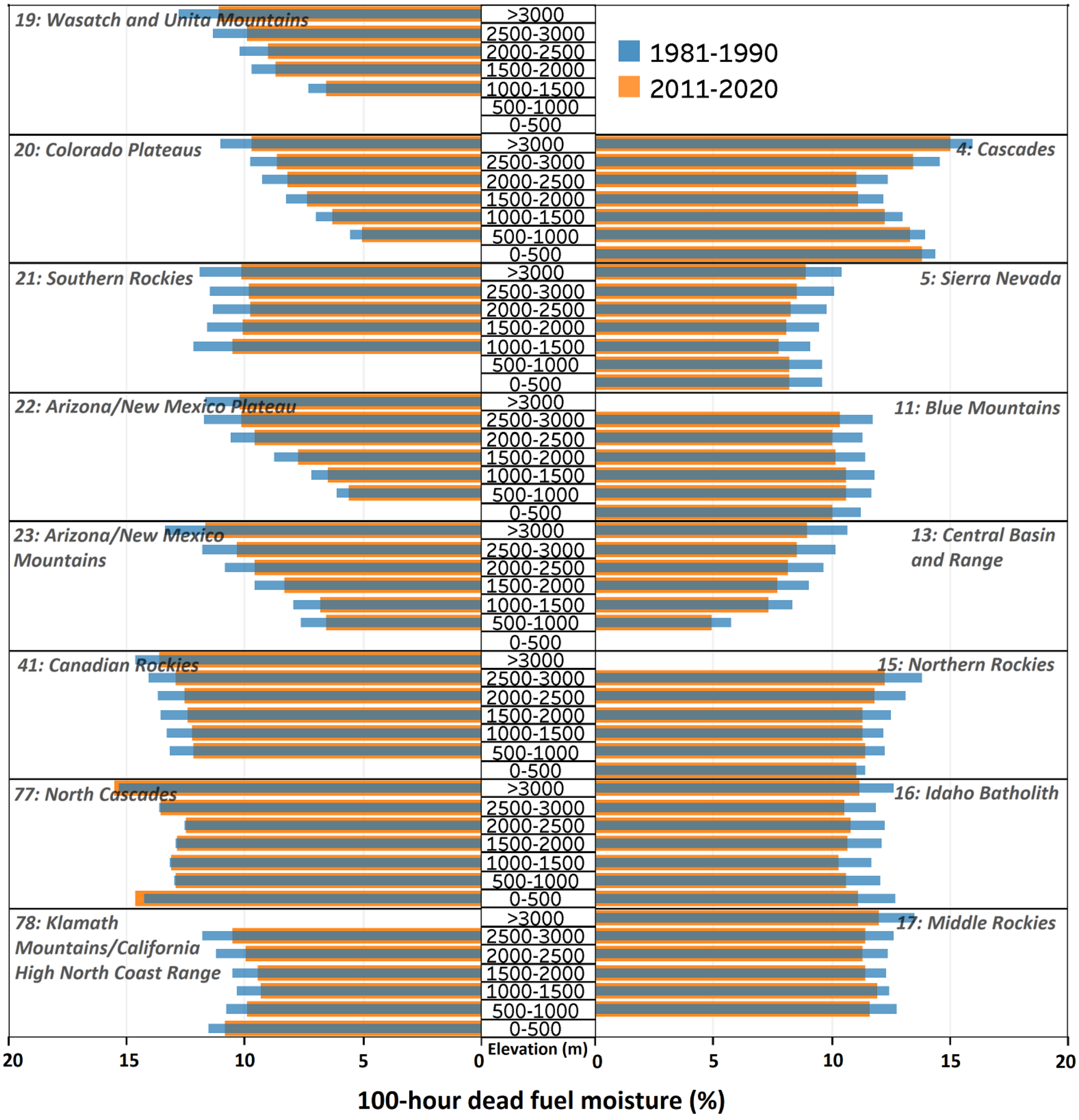

Figure S11. Decadal average warm-season FM100 in each elevation band in each ecoregion. *Results for 1981-1990 and 2011-2020 are shown in blue and orange colors, respectively.*

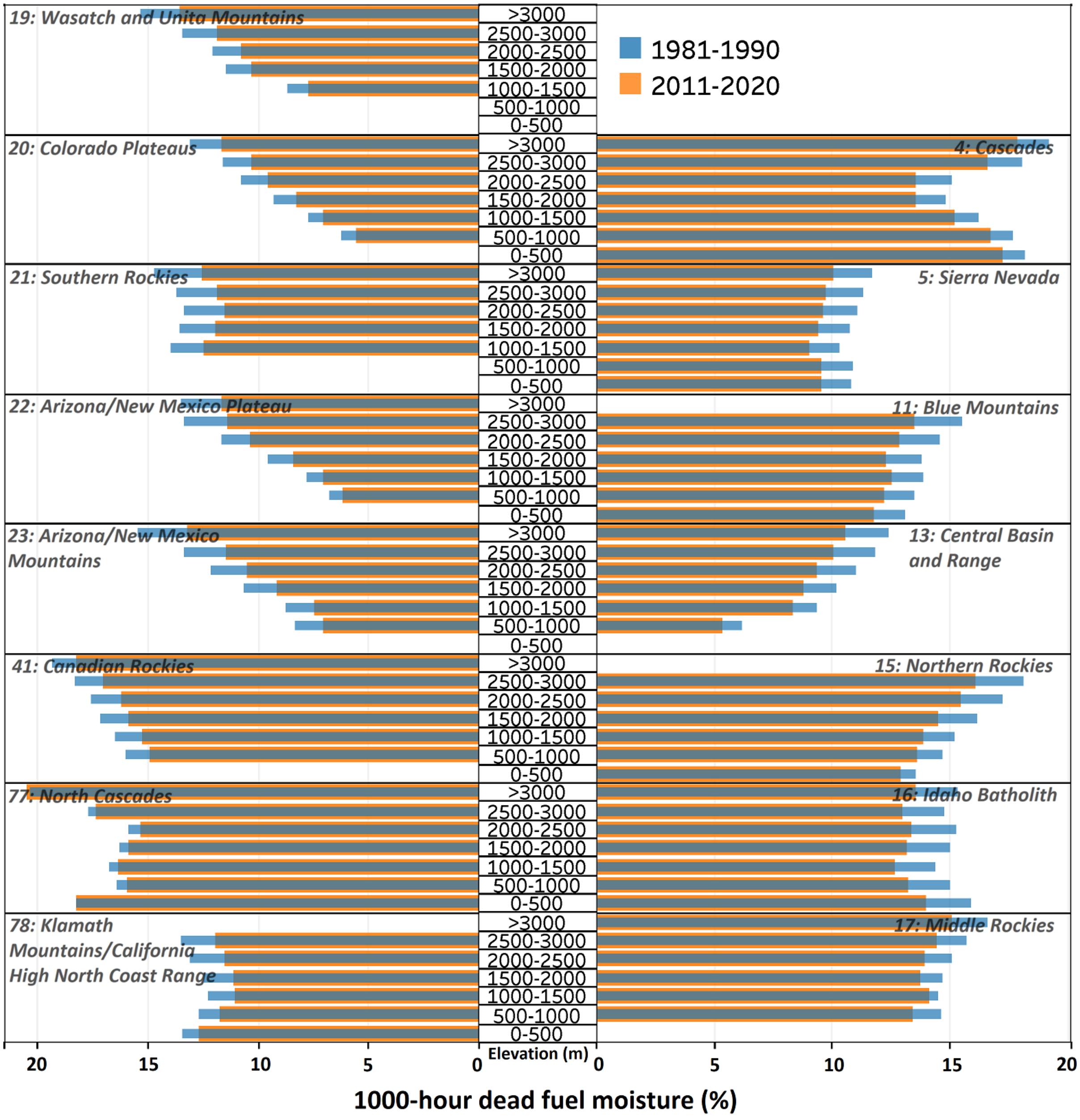

Figure S12. Decadal average warm-season FM1000 in each elevation band in each ecoregion. *Results for 1981-1990 and 2011-2020 are shown in blue and orange colors, respectively.*

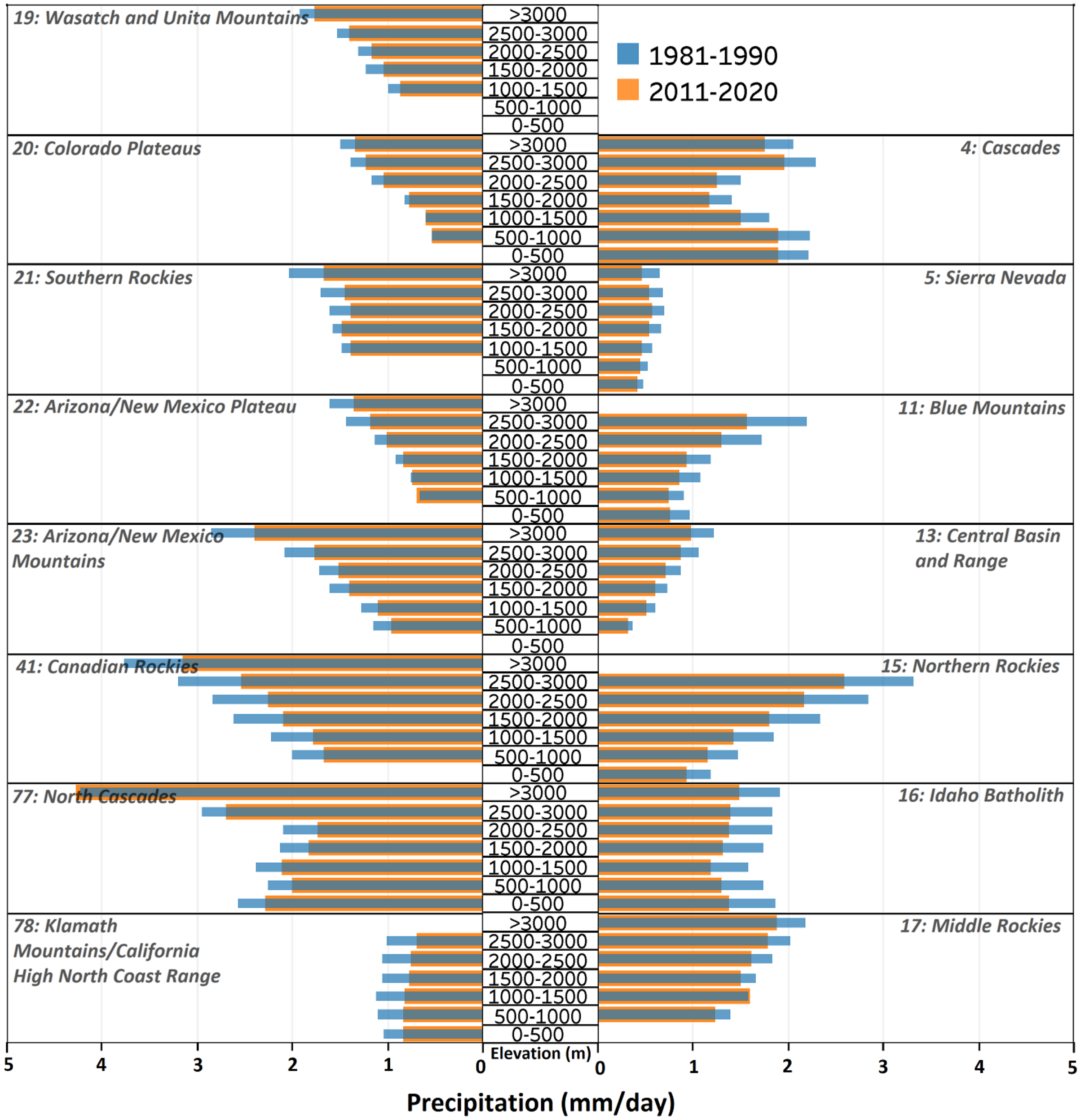

Figure S13. Decadal average warm-season precipitation in each elevation band in each ecoregion. *Results for 1981-1990 and 2011-2020 are shown in blue and orange colors, respectively.*

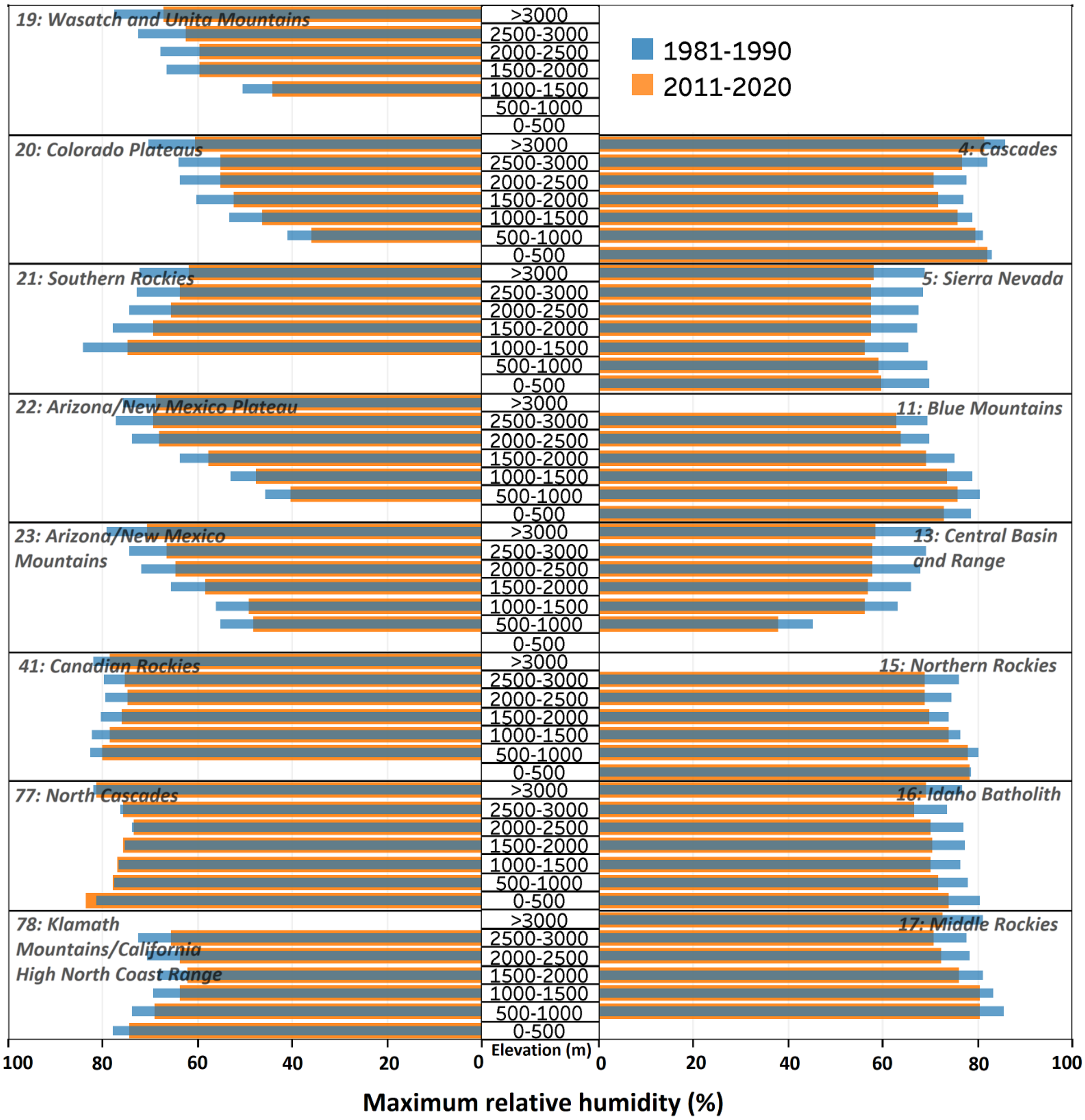

Figure S14. Decadal average warm-season maximum daily relative humidity in each elevation band in each ecoregion. *Results for 1981-1990 and 2011-2020 are shown in blue and orange colors, respectively.*

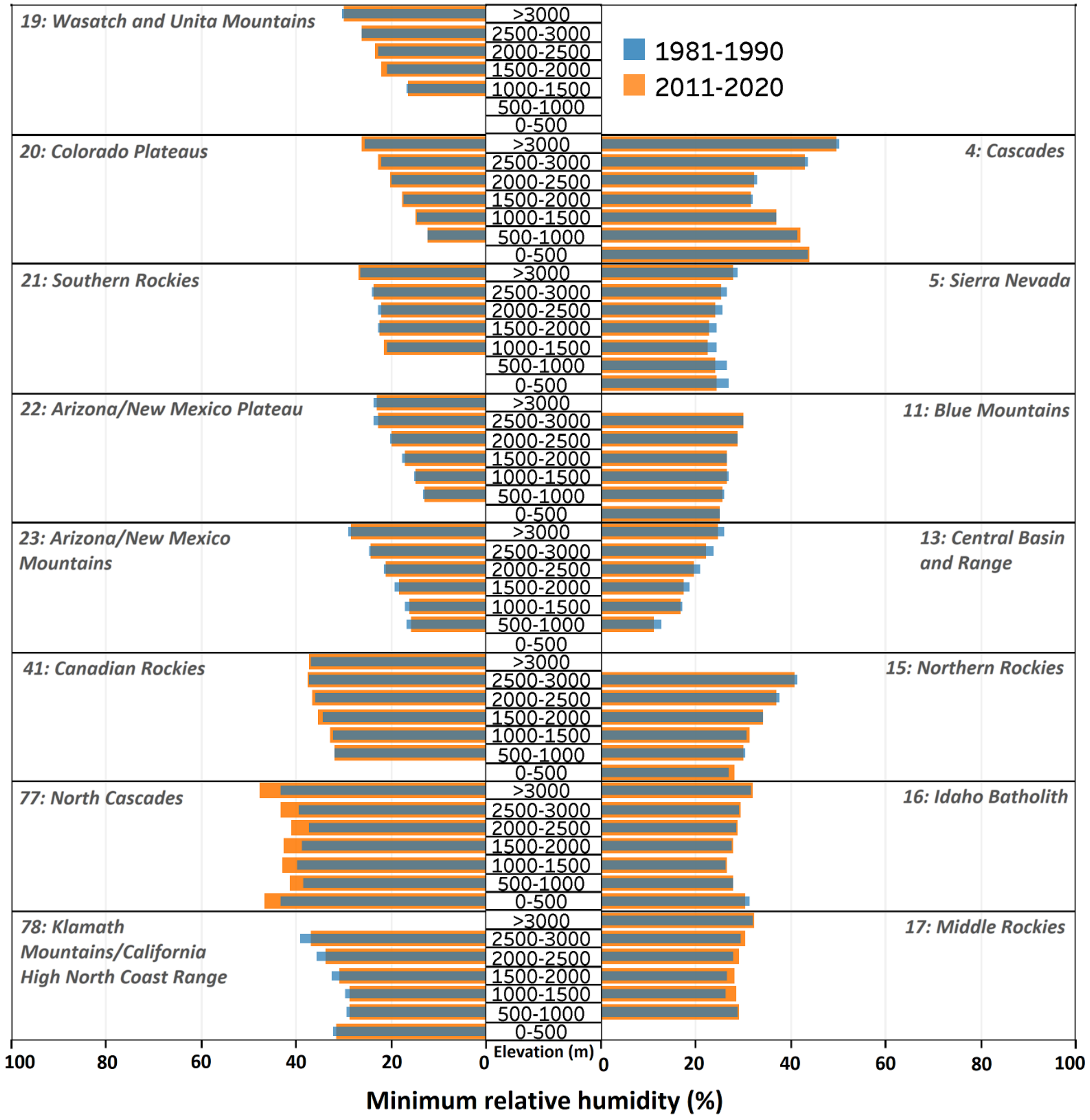

Figure S15. Decadal average warm-season minimum daily relative humidity in each elevation band in each ecoregion. *Results for 1981-1990 and 2011-2020 are shown in blue and orange colors, respectively.*

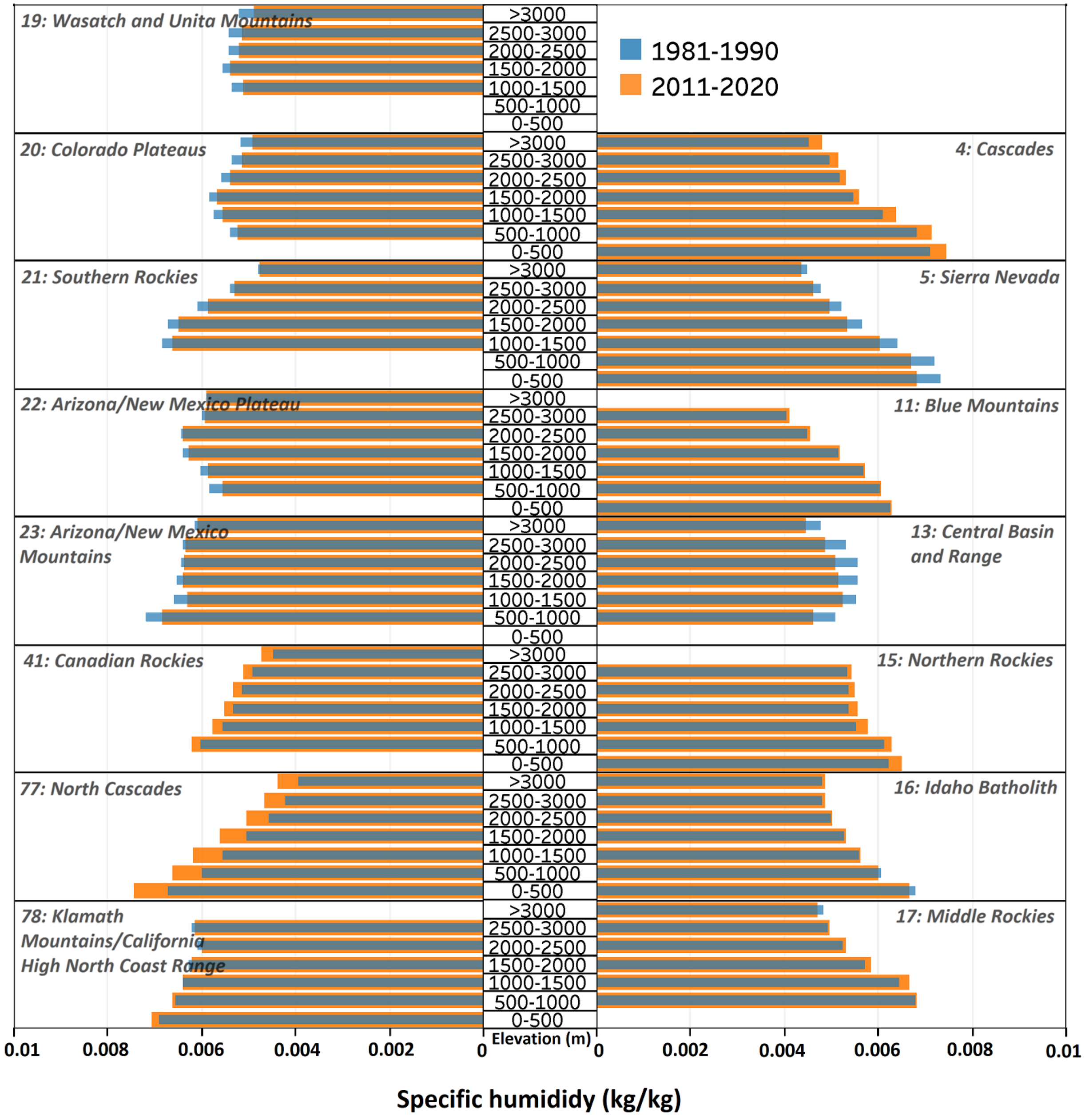

Figure S16. Decadal average warm-season specific humidity in each elevation band in each ecoregion. *Results for 1981-1990 and 2011-2020 are shown in blue and orange colors, respectively.*

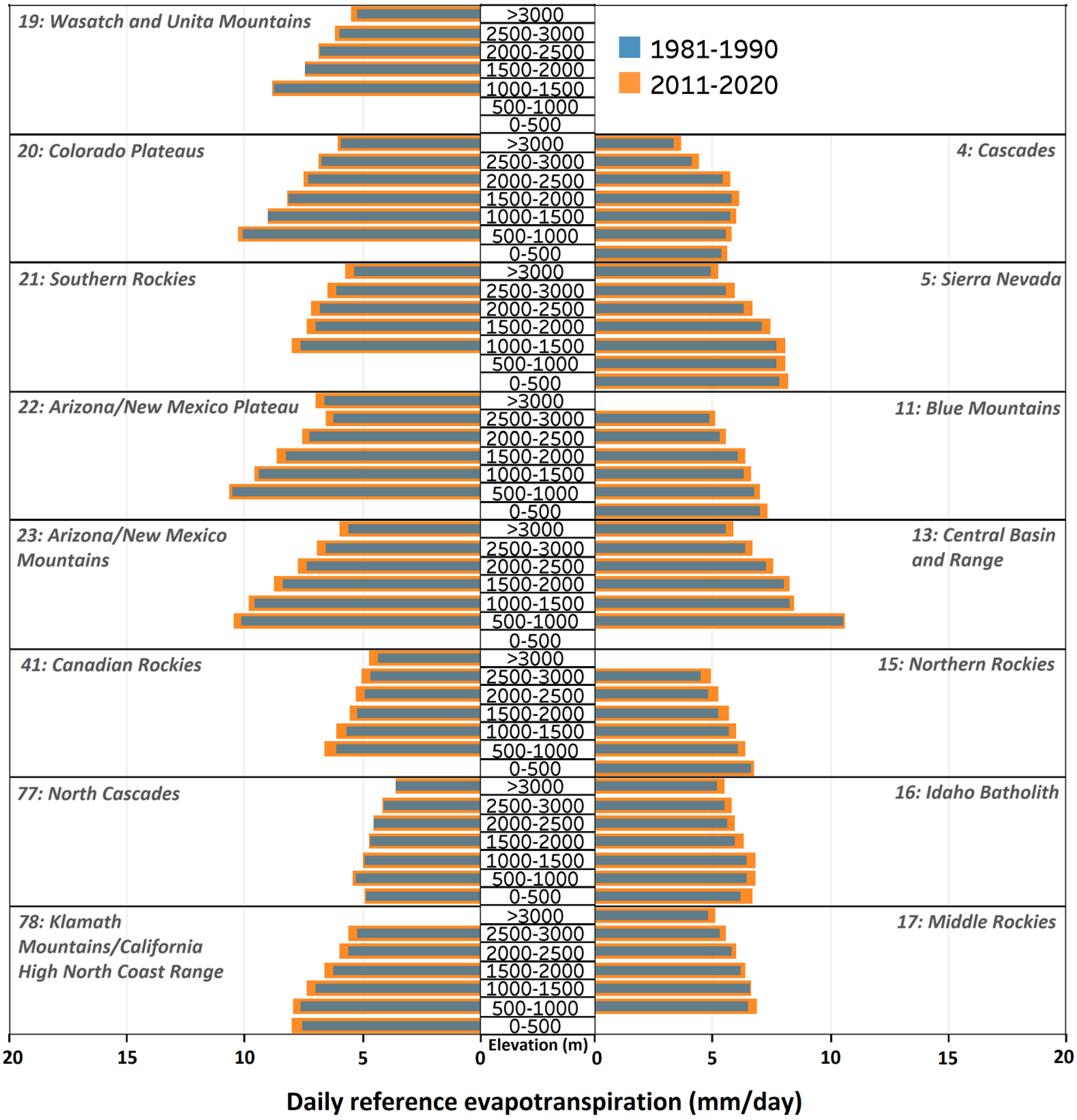

Figure S17. Decadal average warm-season daily reference evapotranspiration in each elevation band in each ecoregion. *Results for 1981-1990 and 2011-2020 are shown in blue and orange colors, respectively.*

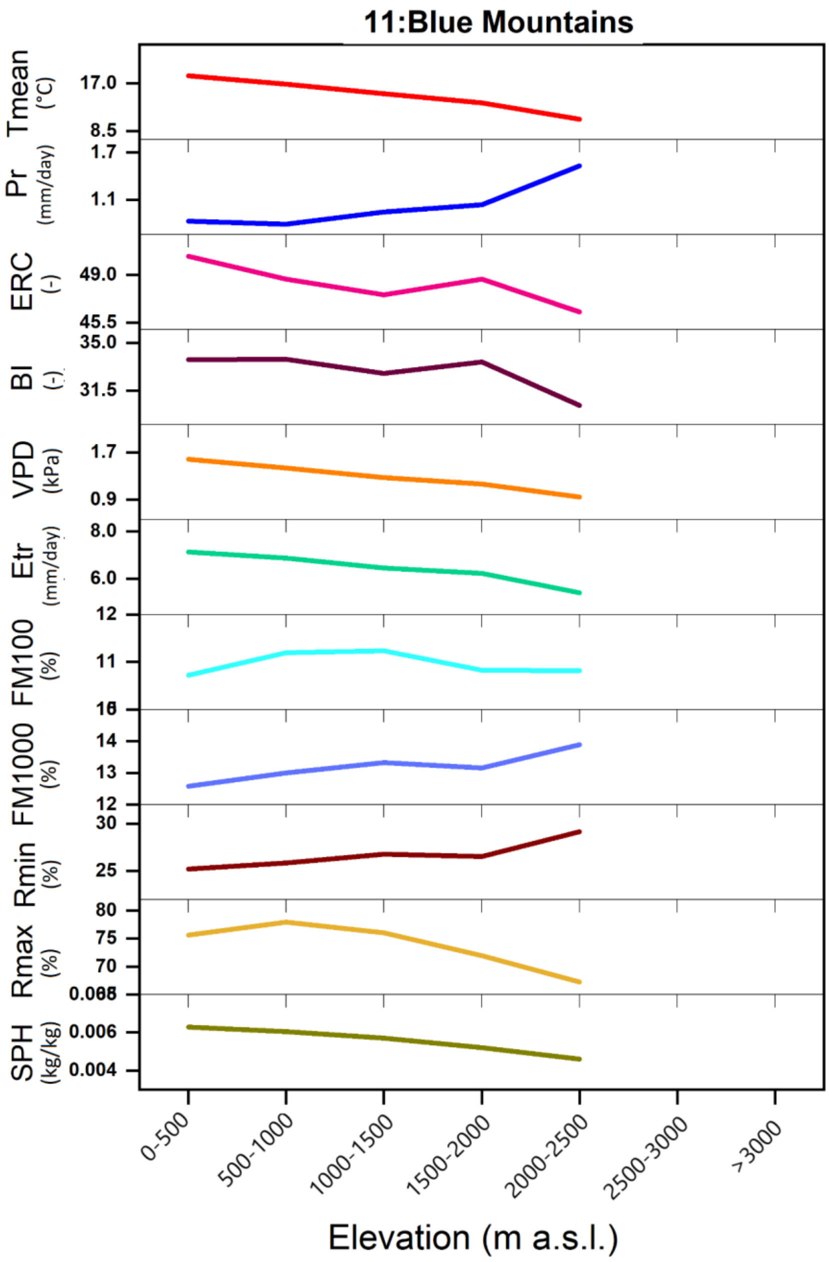

Figure S18. Elevational changes in climatology of meteorological variables and fire danger indices in ecoregion 11: Blue Mountains.

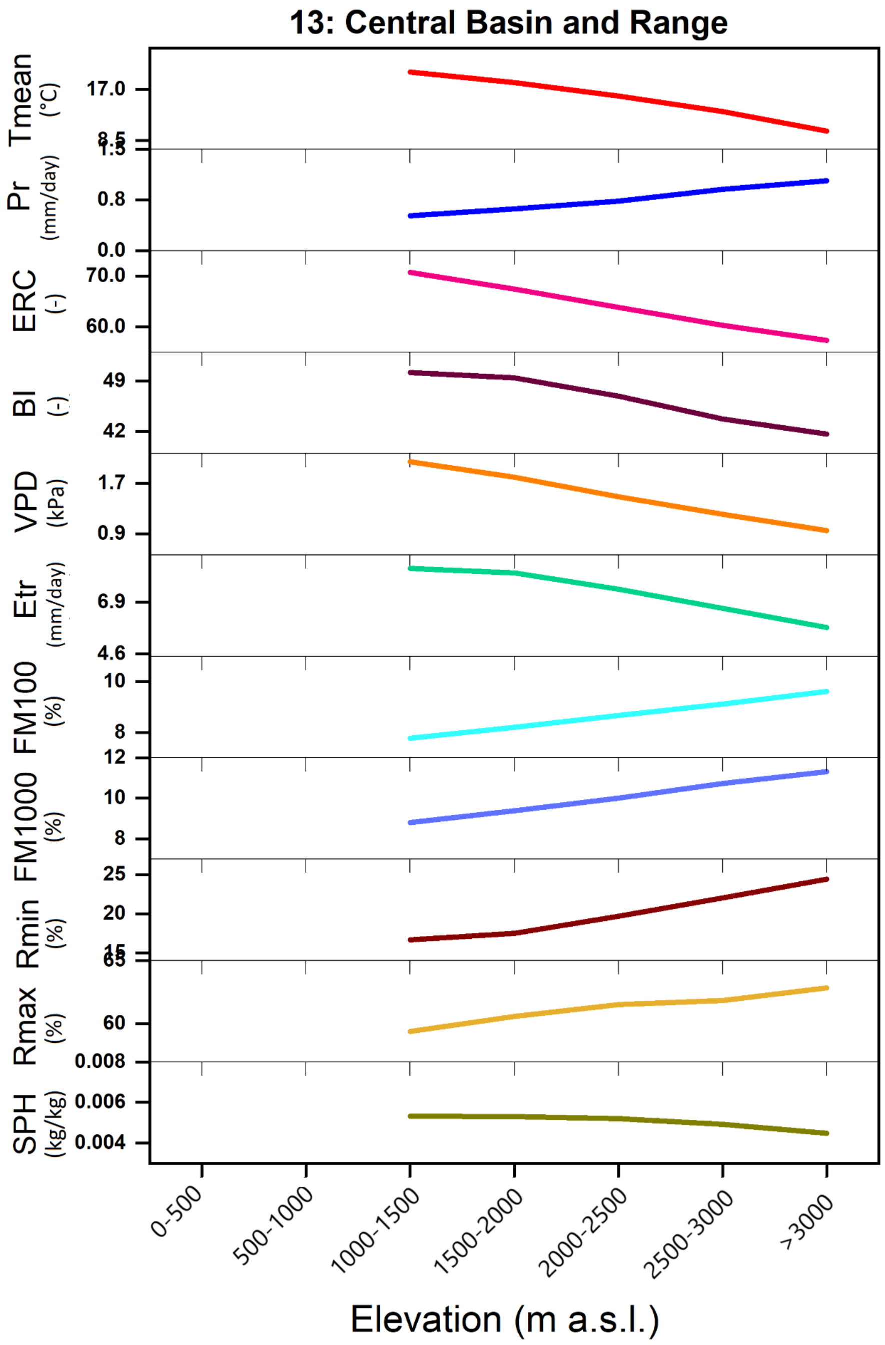

Figure S19. Elevational changes in climatology of meteorological variables and fire danger indices in ecoregion 13: Central Basin and Range.

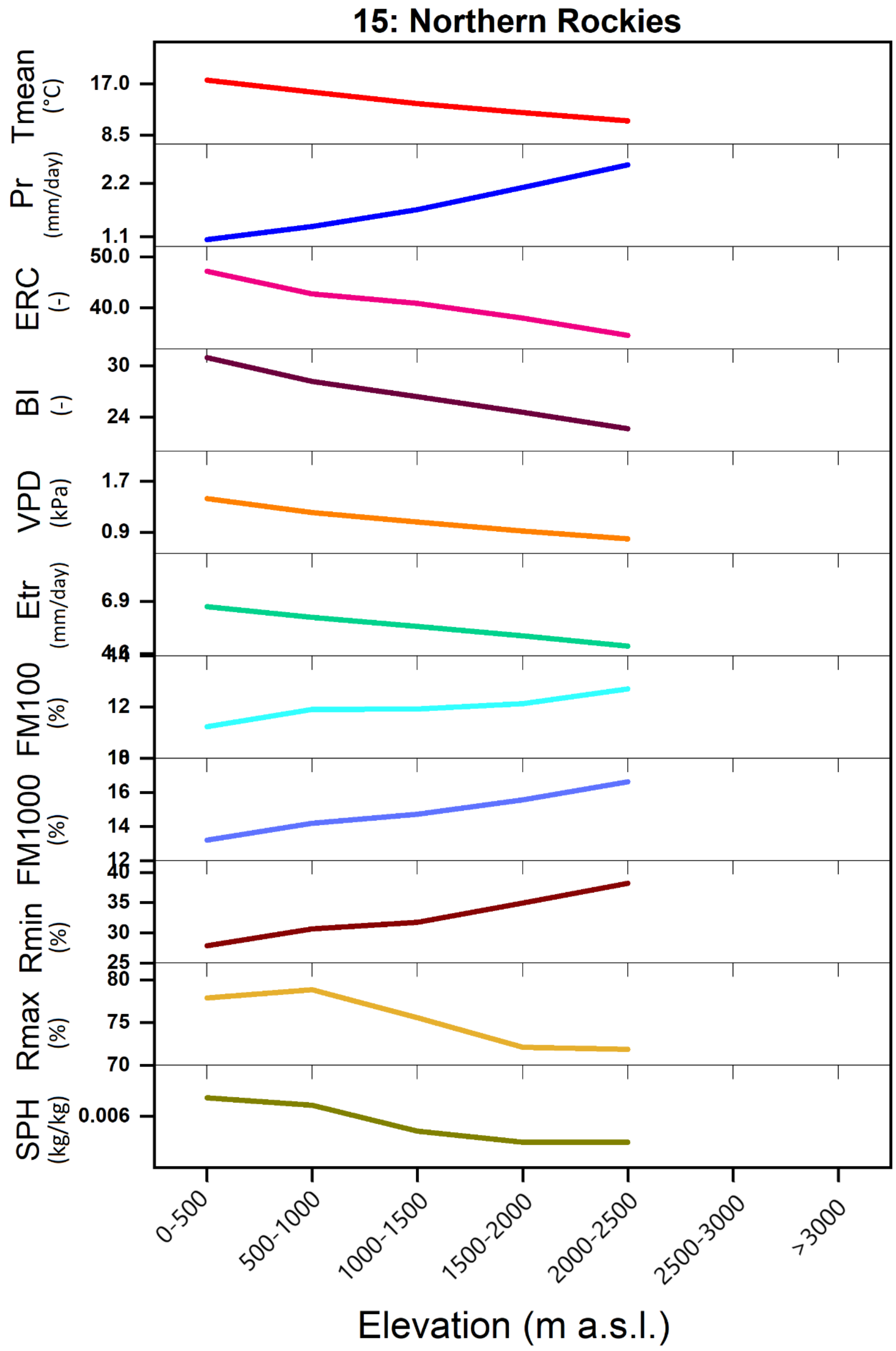

Figure S20. Elevational changes in climatology of meteorological variables and fire danger indices in ecoregion 15: Northern Rockies.

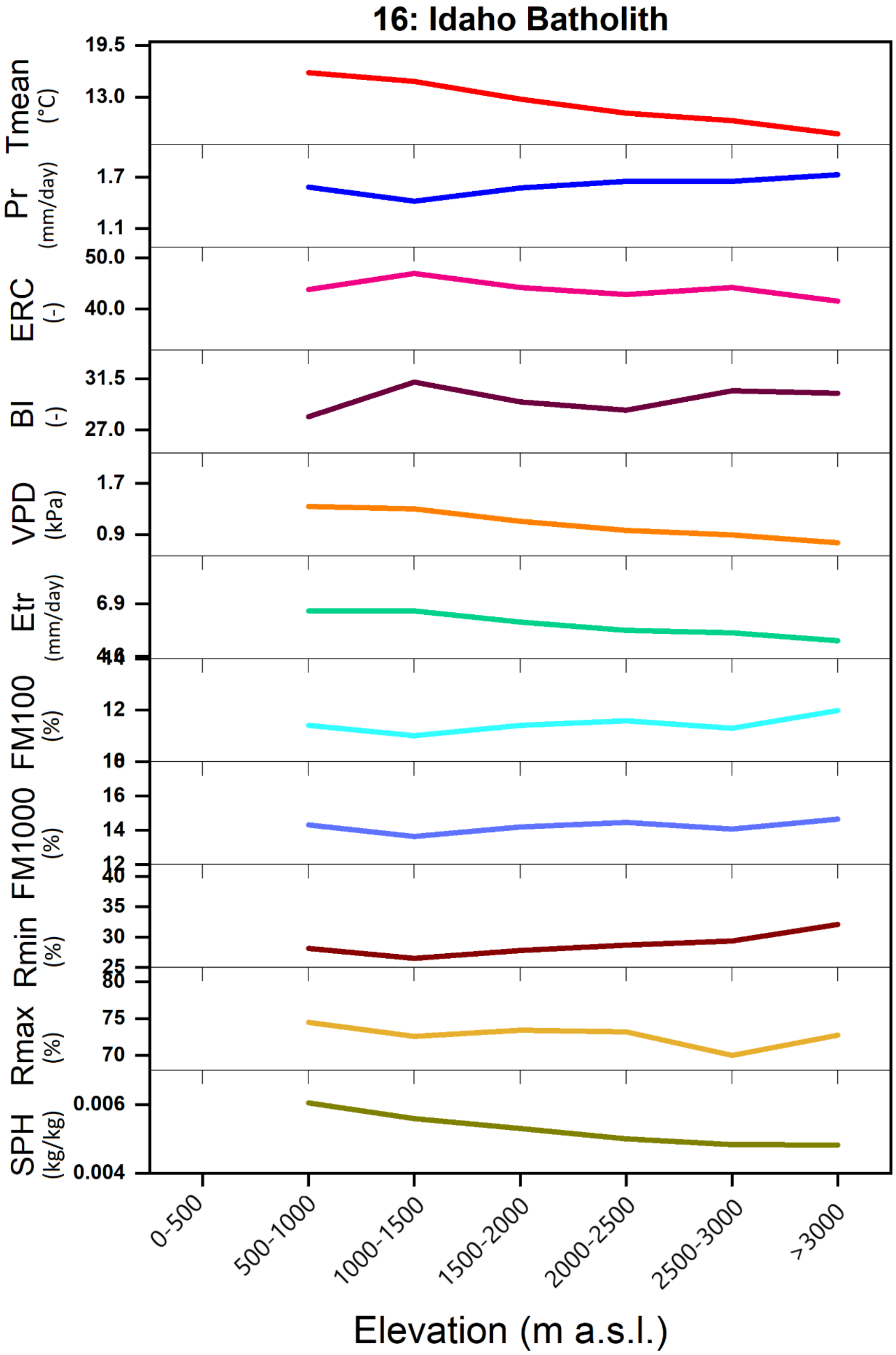

Figure S21. Elevational changes in climatology of meteorological variables and fire danger indices in ecoregion 16: Idaho Batholith.

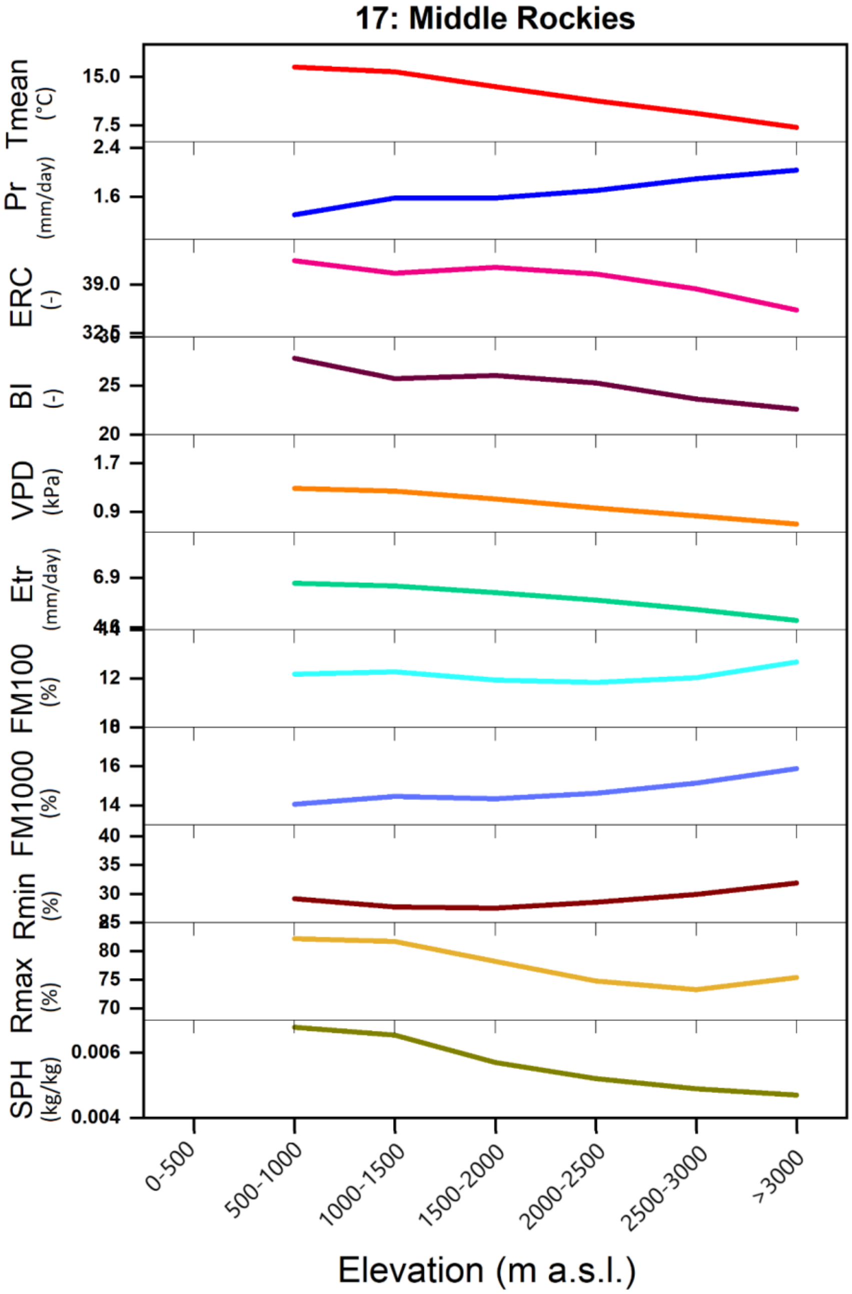

Figure S22. Elevational changes in climatology of meteorological variables and fire danger indices in ecoregion 17: Middle Rockies.

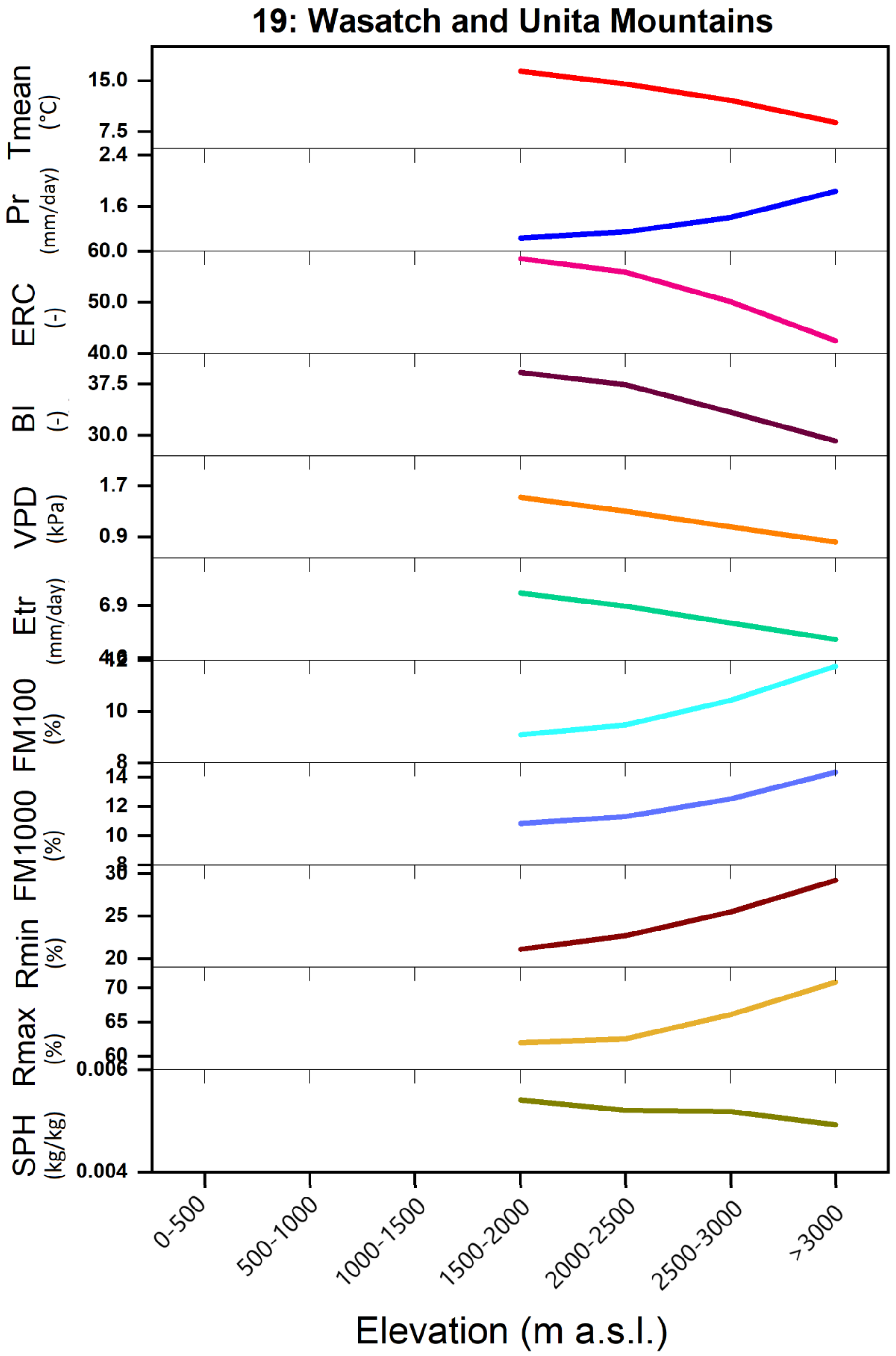

Figure S23. Elevational changes in climatology of meteorological variables and fire danger indices in ecoregion 19: Wasatch and Unita Mountains.

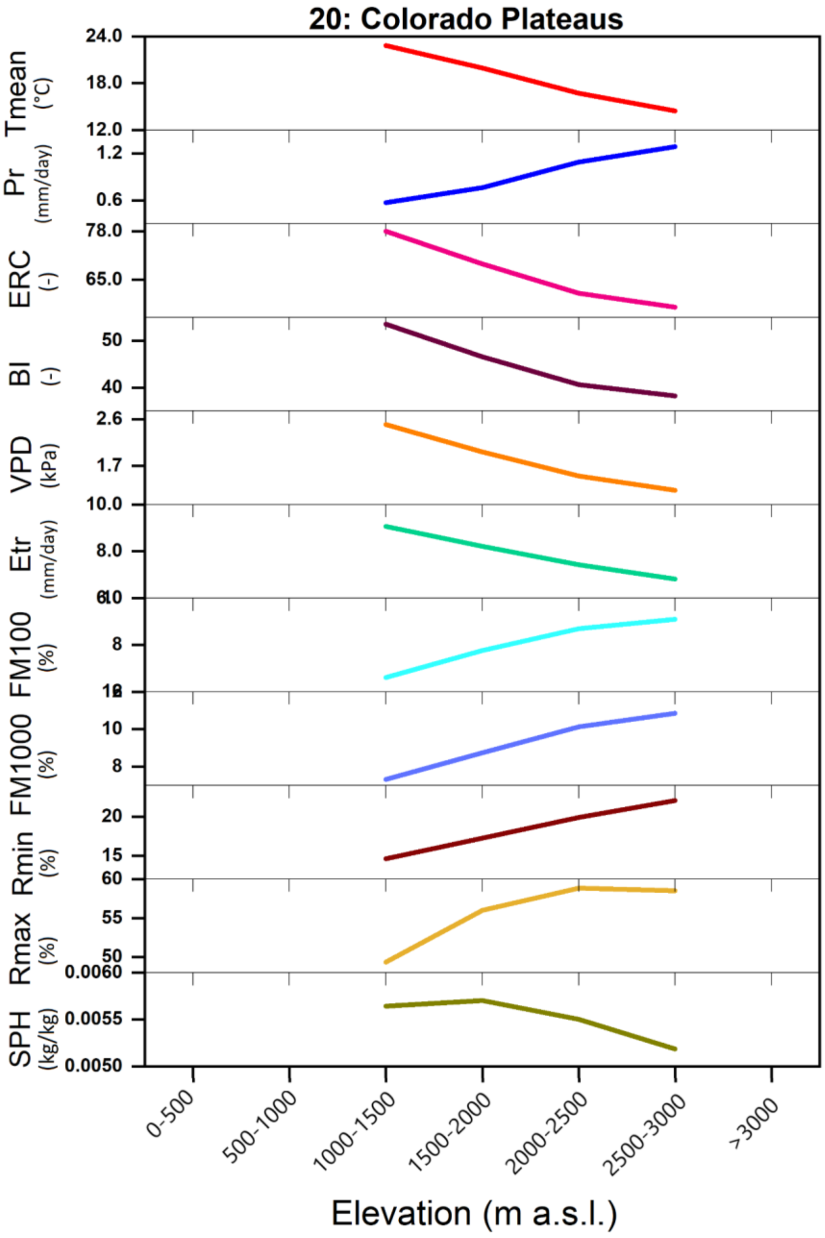

Figure S24. Elevational changes in climatology of meteorological variables and fire danger indices in ecoregion 20: Colorado Plateau.

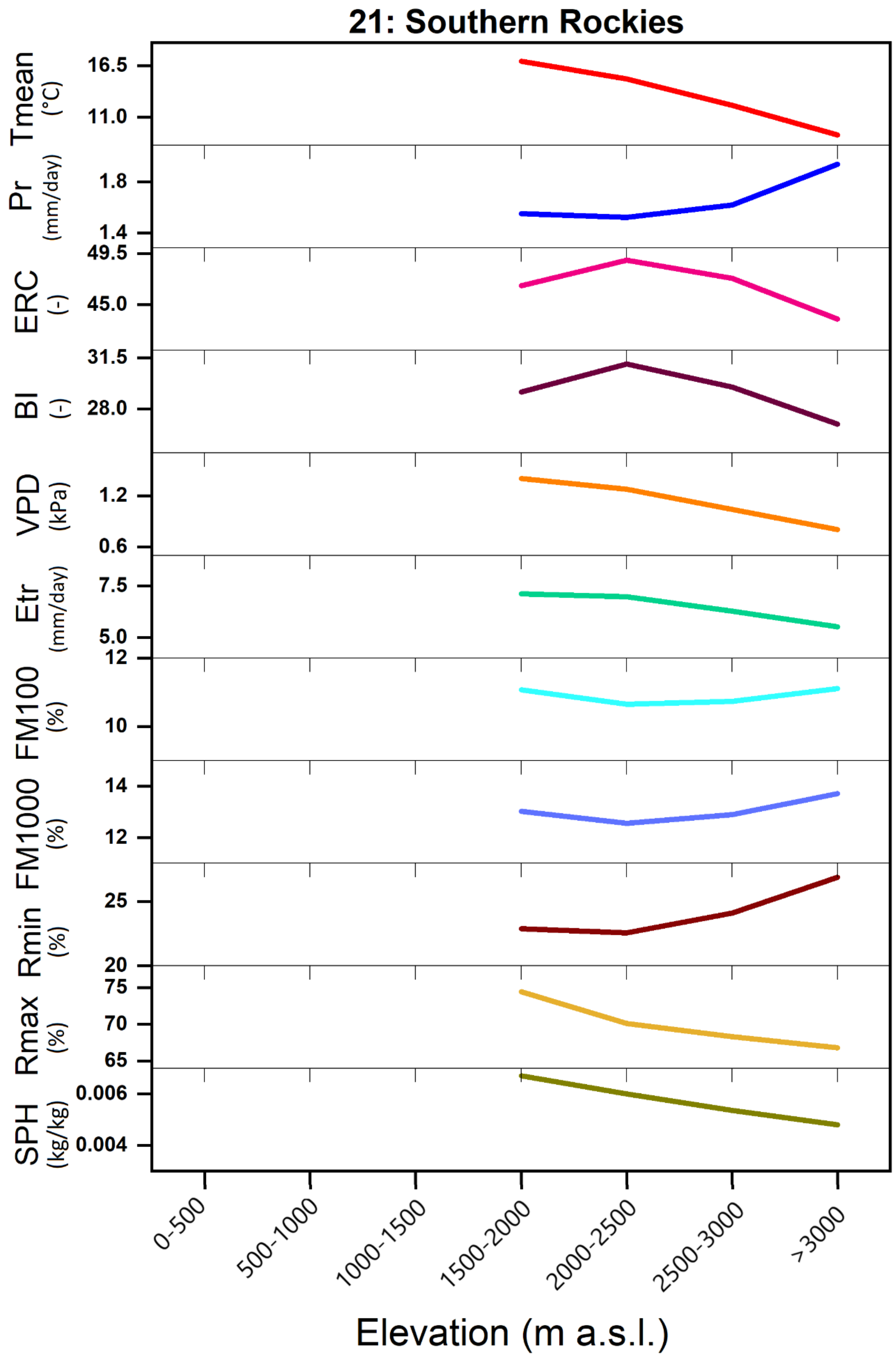

Figure S25. Elevational changes in climatology of meteorological variables and fire danger indices in ecoregion 21: Southern Rockies.

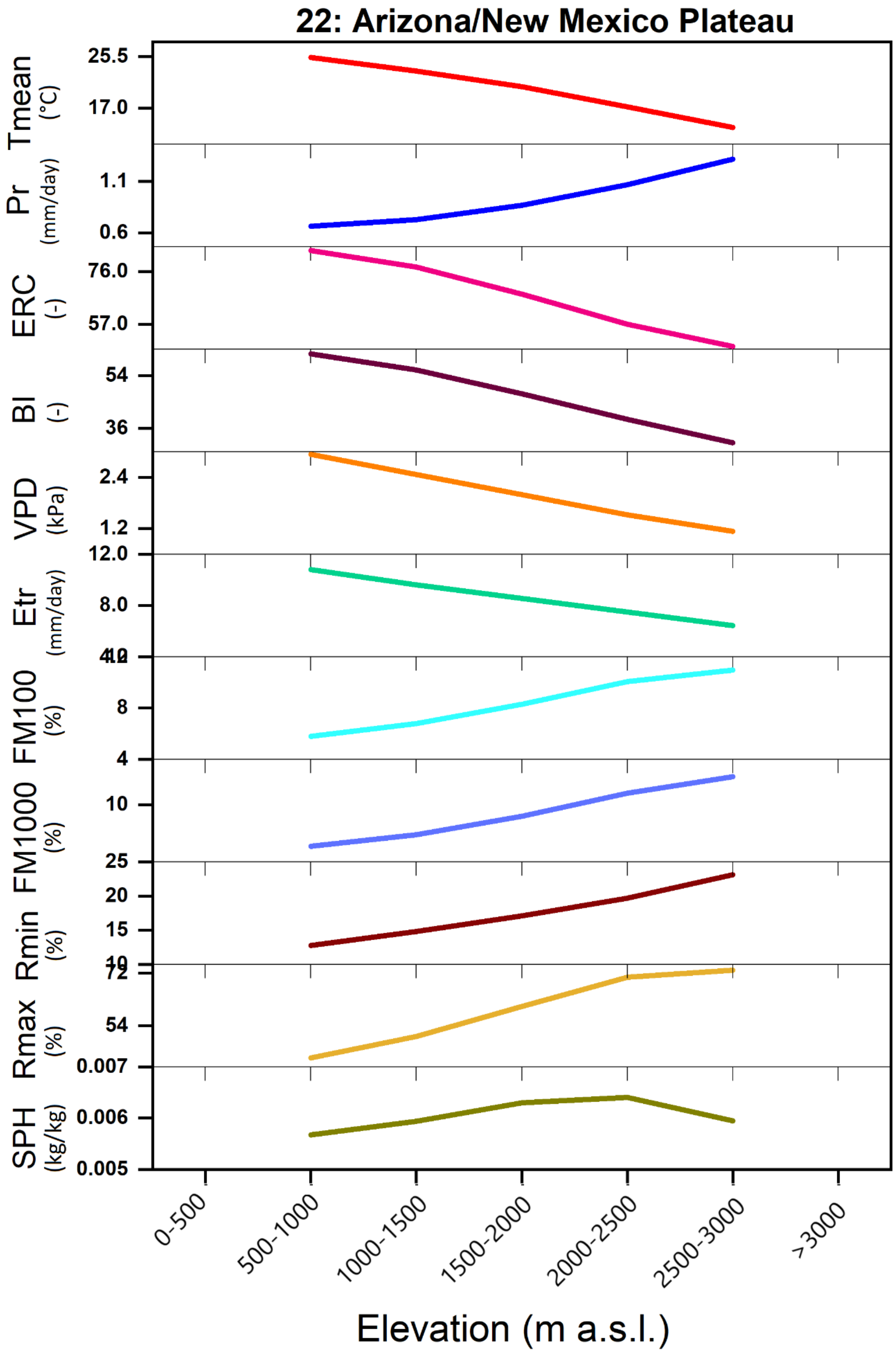

Figure S26. Elevational changes in climatology of meteorological variables and fire danger indices in ecoregion 22: Arizona/New Mexico Plateau.

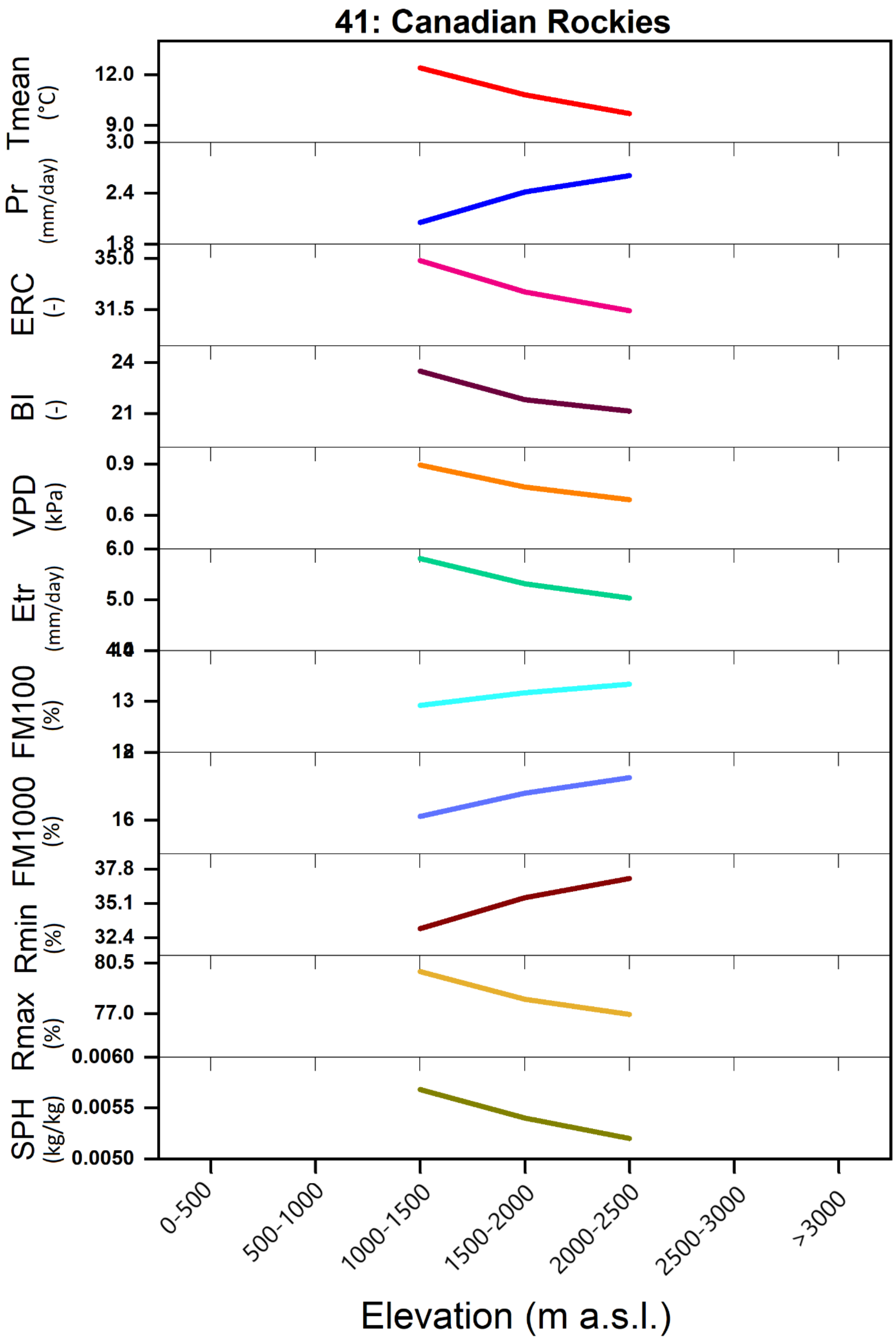

Figure S27. Elevational changes in climatology of meteorological variables and fire danger indices in ecoregion 41: Canadian Rockies.

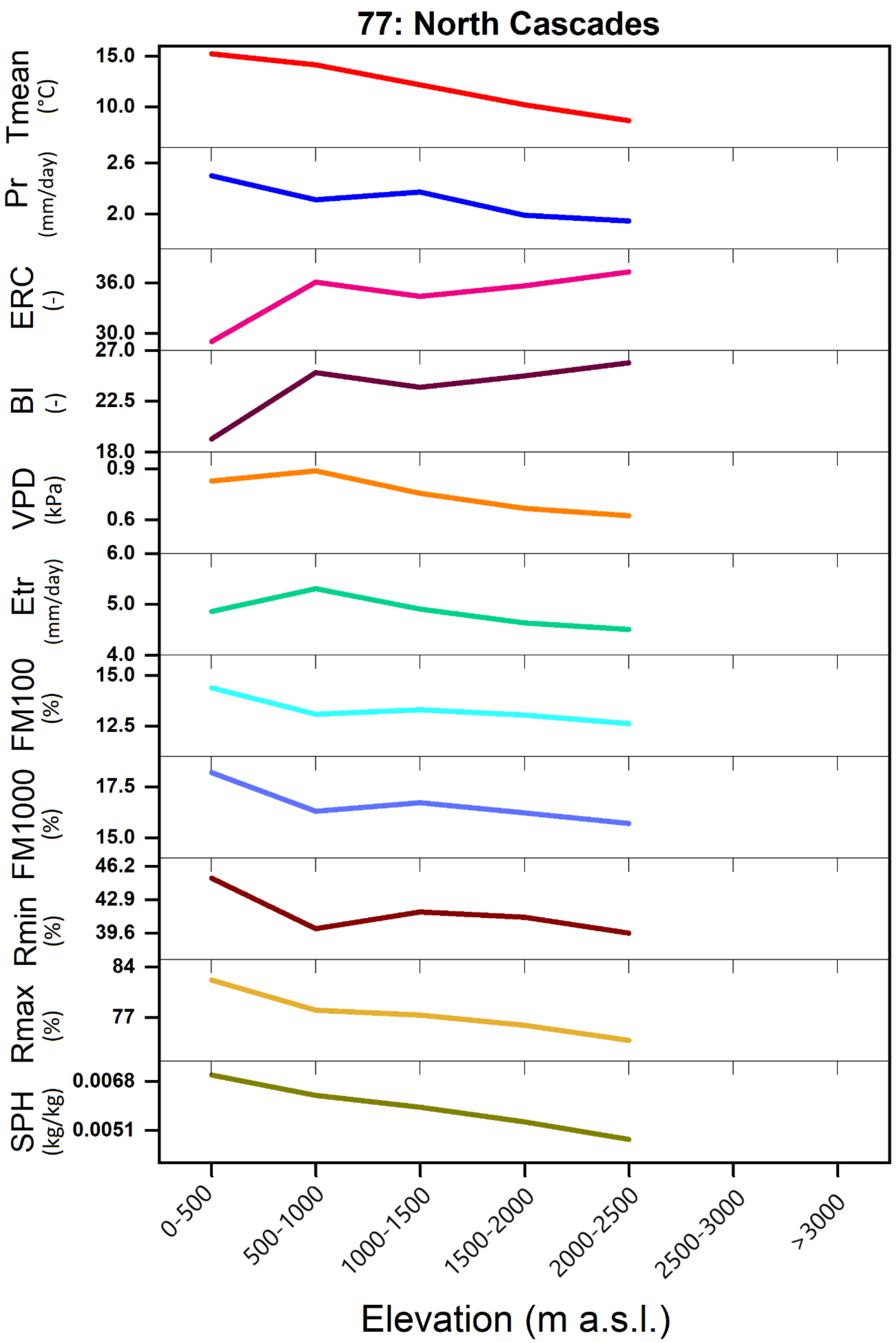

Figure S28. Elevational changes in climatology of meteorological variables and fire danger indices in ecoregion 77: North Cascades.

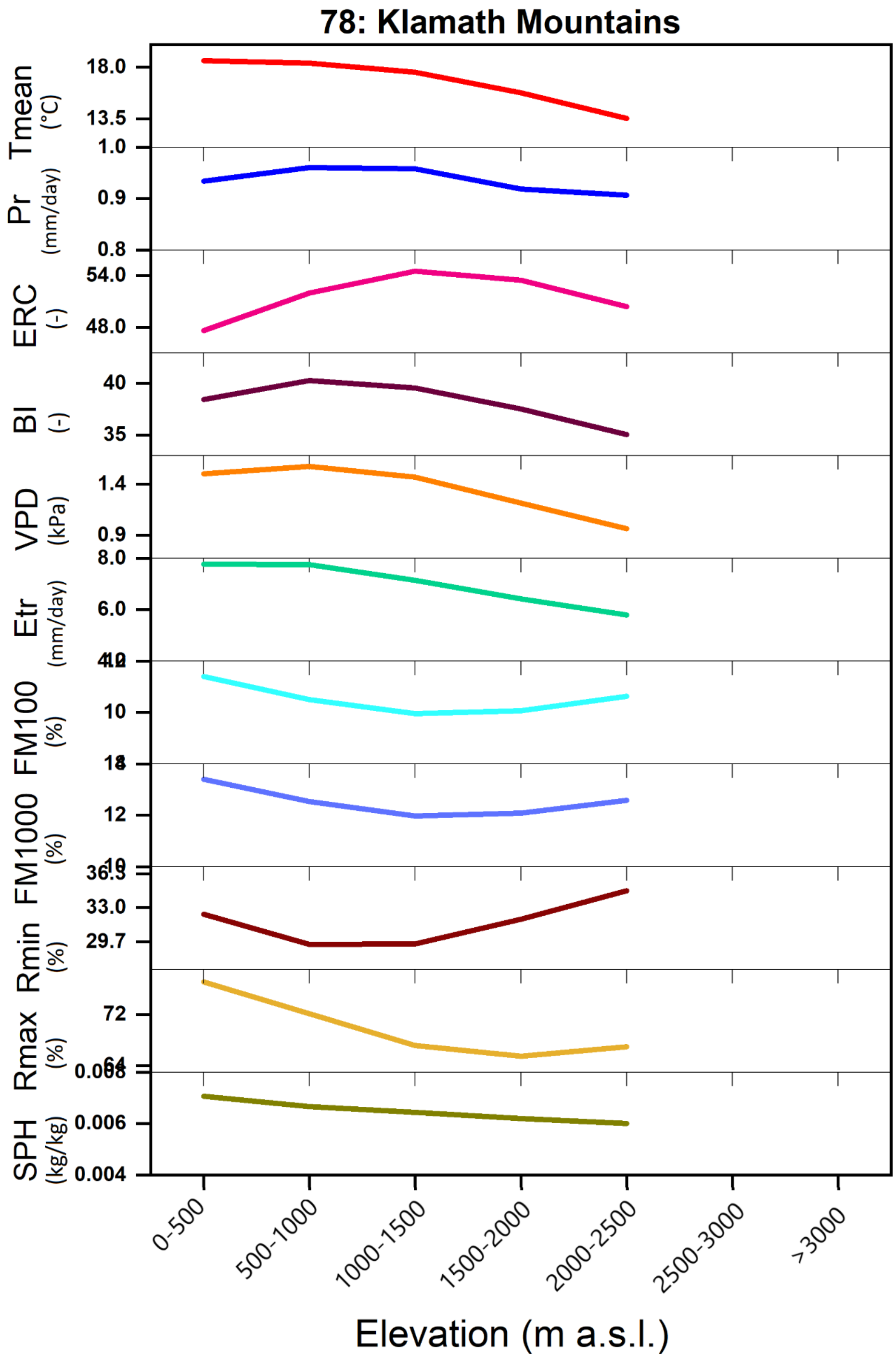

Figure S29. Elevational changes in climatology of meteorological variables and fire danger indices in ecoregion 78: Klamath Mountains/California High North Coast Range.

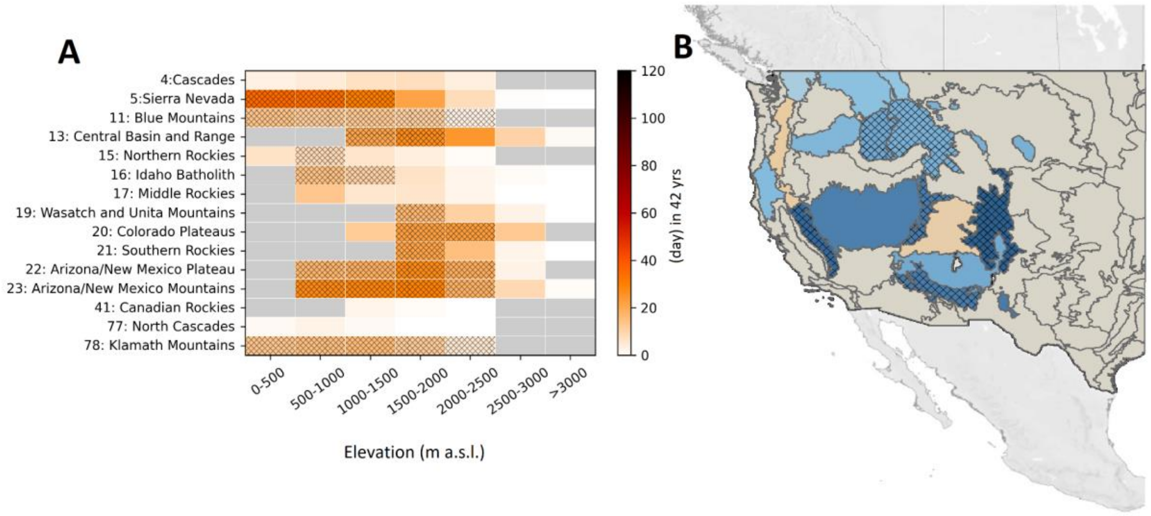

Figure S30. Elevation-dependent increase of critical fire danger days (VPD≥2 kPa). (A) *Temporal trends in critical fire danger days from 1979-2020 in each elevation band and ecoregion.* ***(B)*** *Slope of temporal trends in critical fire danger days across elevation bands. (© OpenStreetMap contributors 2017. Distributed under the Open Data Commons Open Database License (ODbL) v1.0.)*[35].

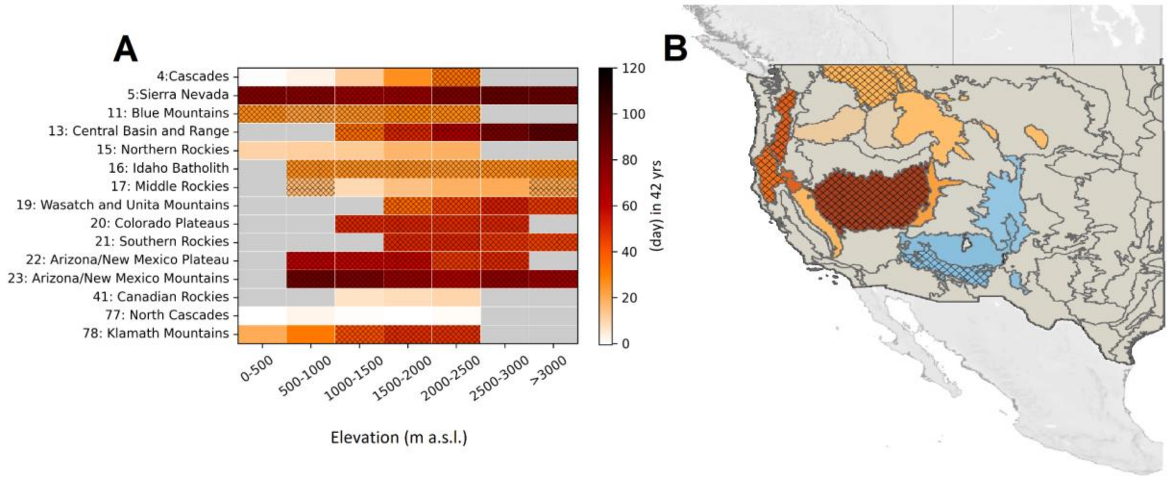

Figure S31. Elevation-dependent increase of critical fire danger days (FM100≤8%). (A) *Temporal trends in critical fire danger days from 1979-2020 in each elevation band and ecoregion.* ***(B)*** *Slope of temporal trends in critical fire danger days across elevation bands. (© OpenStreetMap contributors 2017. Distributed under the Open Data Commons Open Database License (ODbL) v1.0.)*[35].

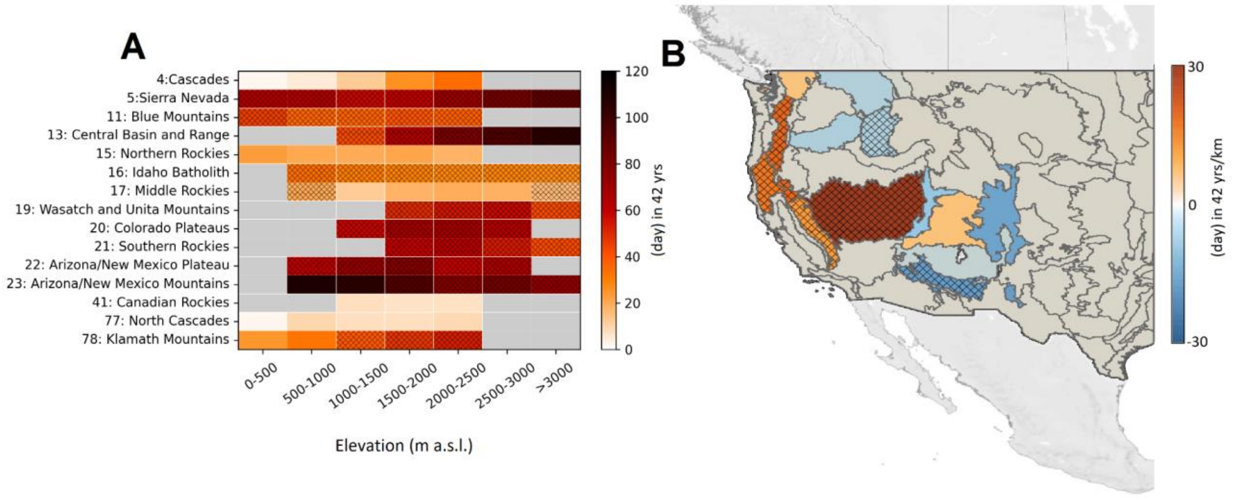

Figure S32. Elevation-dependent increase of critical fire danger days (FM1000$\leq$10%). (A) *Temporal trends in critical fire danger days from 1979-2020 in each elevation band and ecoregion.* ***(B)*** *Slope of temporal trends in critical fire danger days across elevation bands. (© OpenStreetMap contributors 2017. Distributed under the Open Data Commons Open Database License (ODbL) v1.0.)*[35].

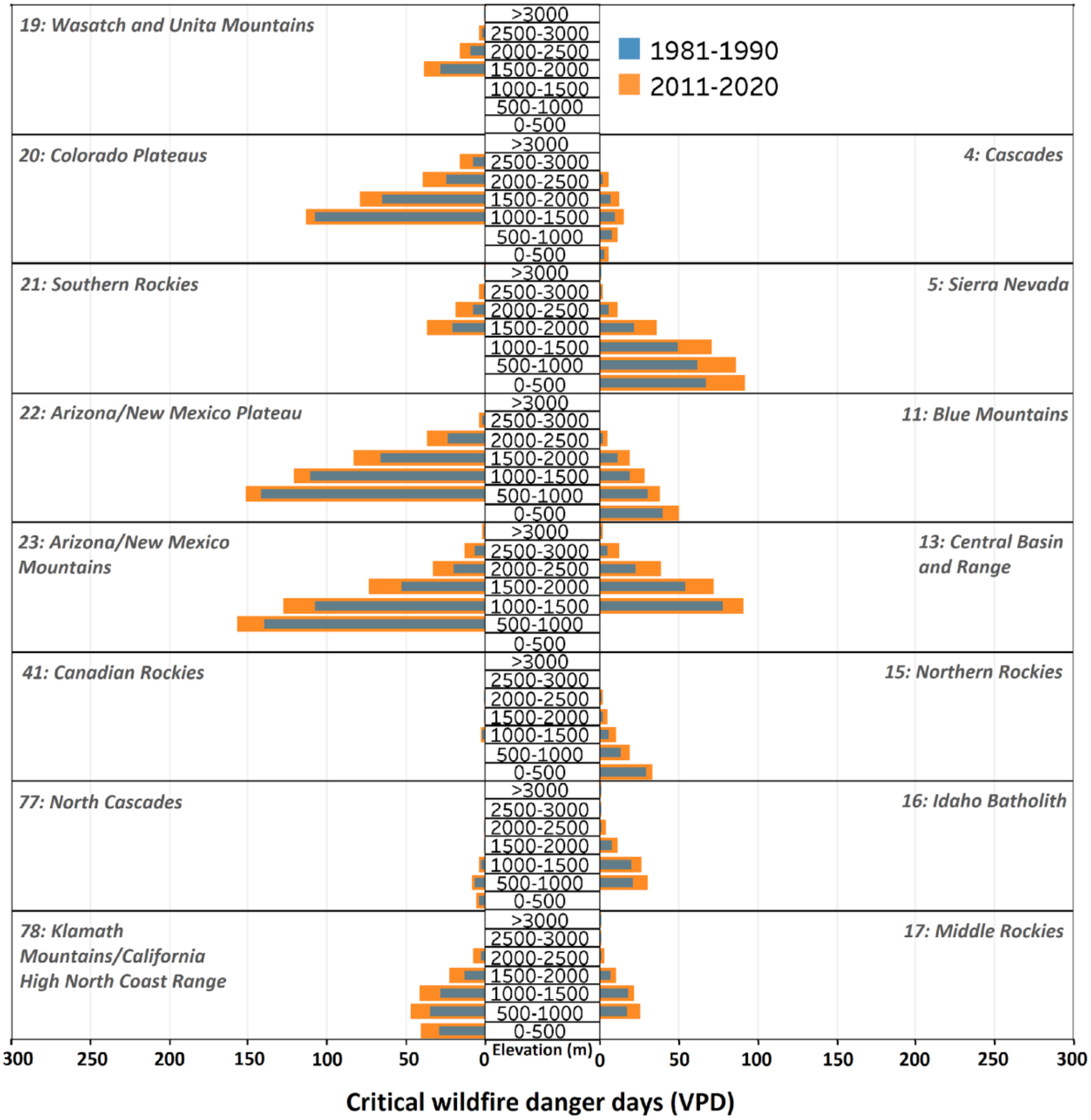

Figure S33. Annual critical fire danger days based on VPD (VPD≥2 kPa) in the first (1981-1990) and last (2011-2020) decades of the study period.

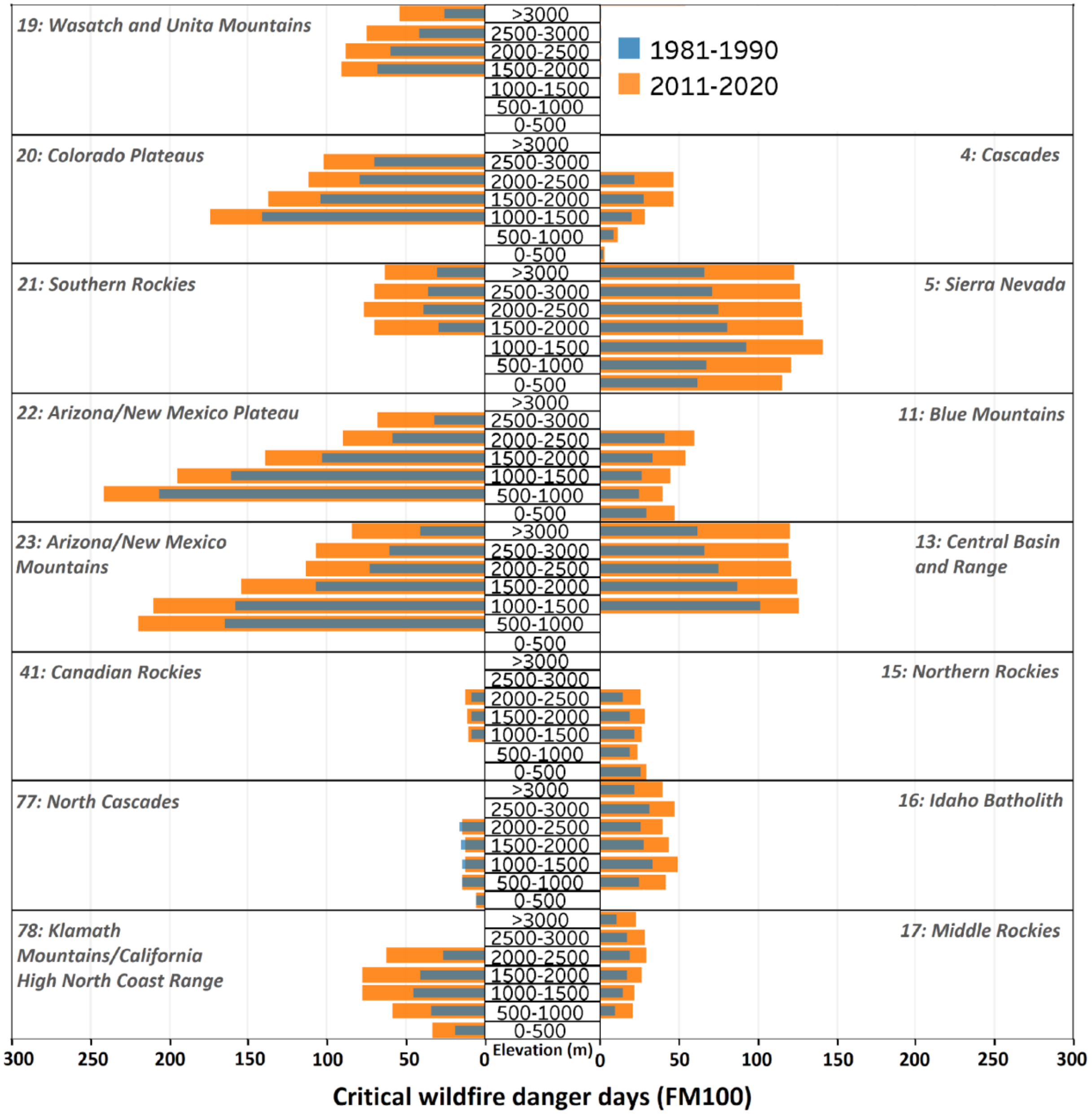

Figure S34. Annual critical fire danger days based on FM100 (FM100≤8%) in the first (1981-1990) and last (2011-2020) decades of the study period.

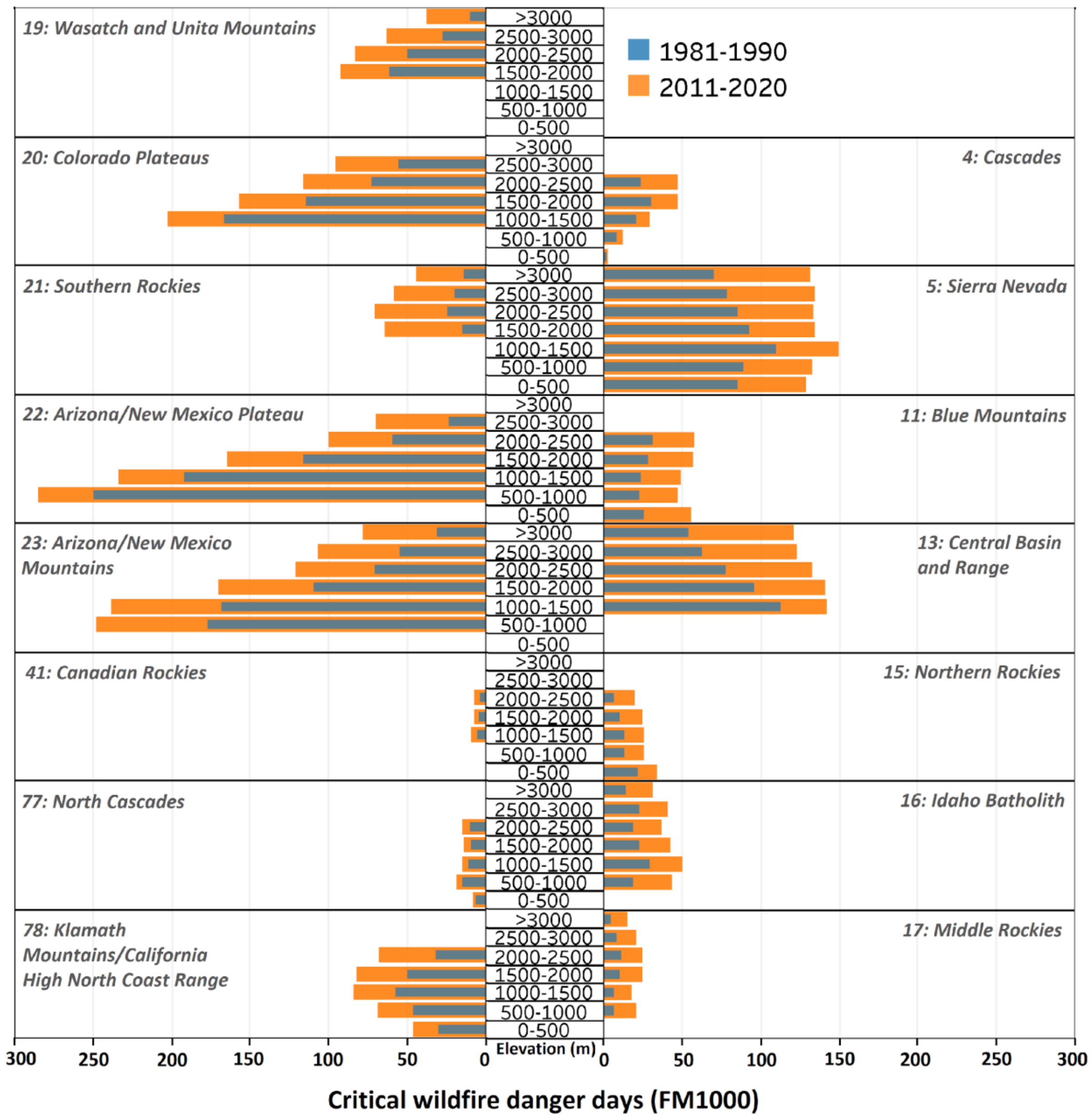

Figure S35. Annual critical fire danger days based on FM1000 (FM1000≤10%) in the first (1981-1990) and last (2011-2020) decades of the study period.

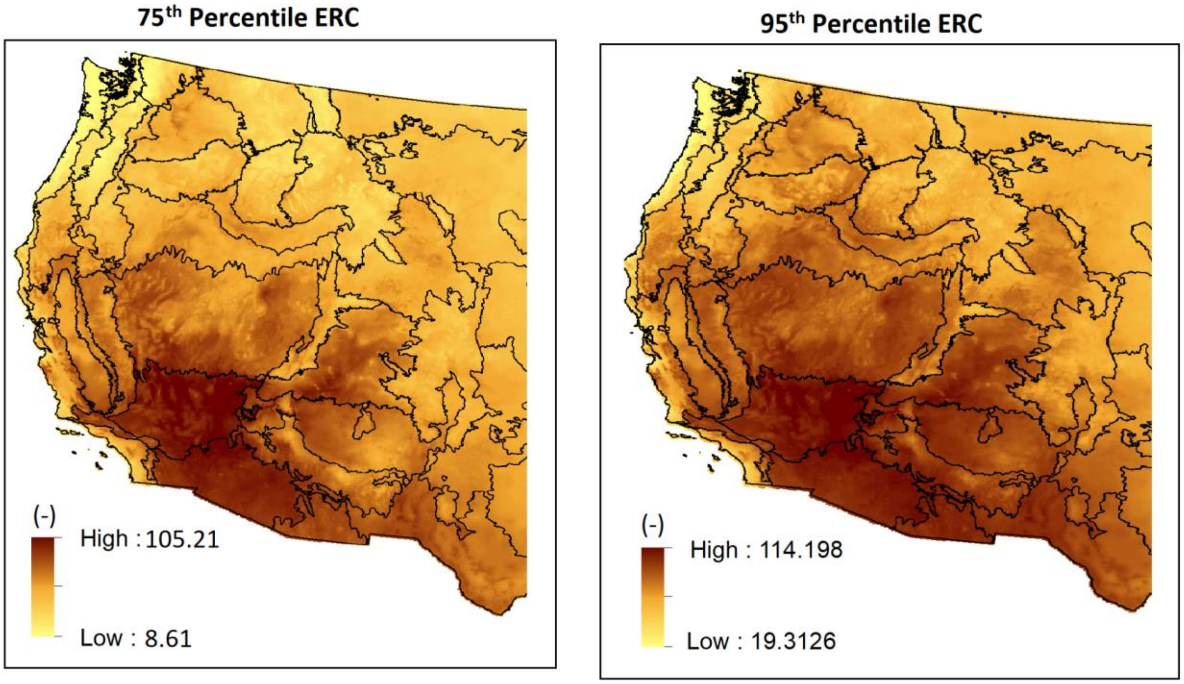

Figure S36. ERC threshold associated with the 75th and 95th percentiles, respectively, of daily ERC values from 1979-2020.

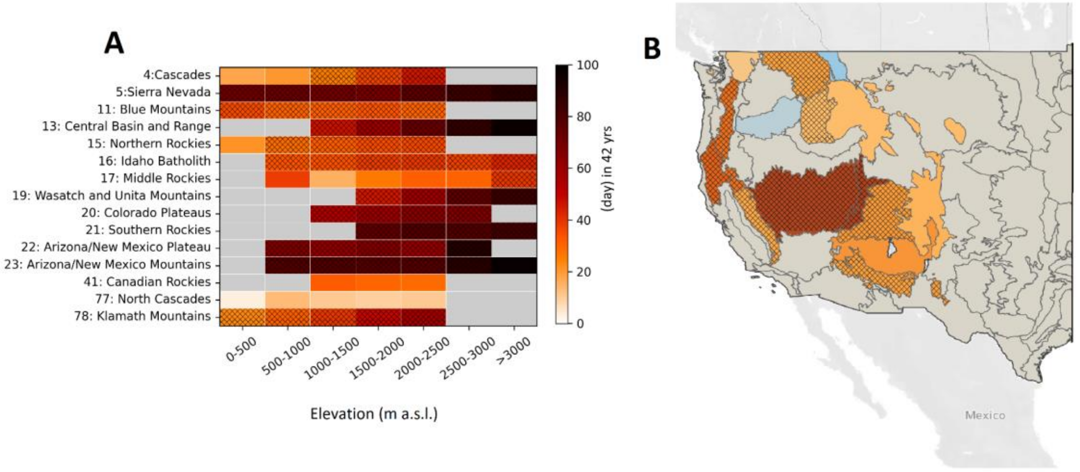

Figure S37. Elevation-dependent increase in high fire danger days. (A) *Temporal trends in high fire danger days – associated with daily ERC larger than the 75th percentile of long-term record from 1979-2020 – in each elevation band and ecoregion.* ***(B)*** *Slope of temporal trends in high fire danger days across elevation bands. Hatched areas indicate statistically significant trends at the 95% confidence level. (© OpenStreetMap contributors 2017. Distributed under the Open Data Commons Open Database License (ODbL) v1.0.)*[35].

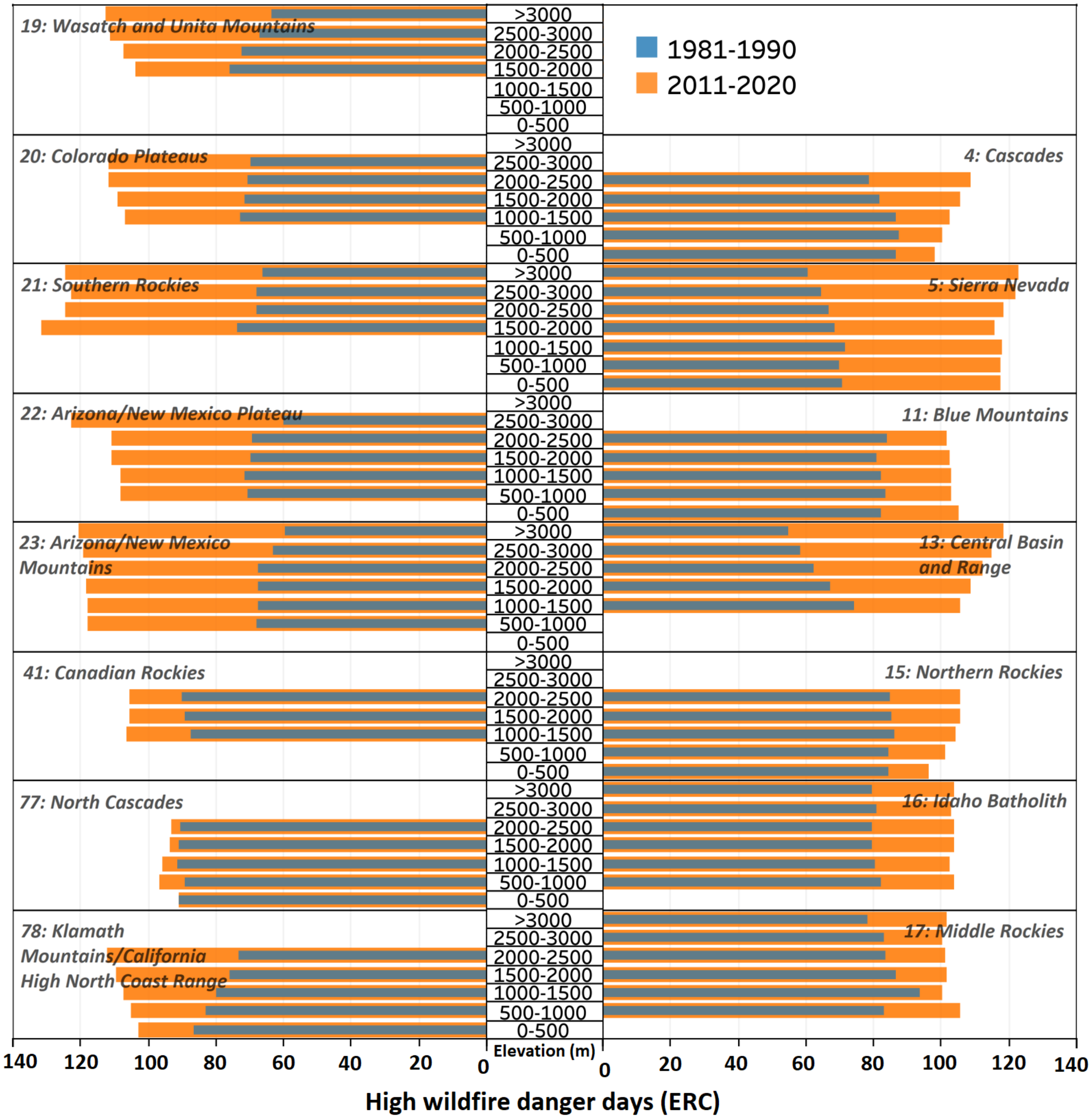

Figure S38. Annual high fire danger days associated with daily ERC larger than the 75th percentile of long-term record from 1979-2020. *Decadal average high fire danger days per year from 1981-1990 (blue) and 2011-2020 (orange).*

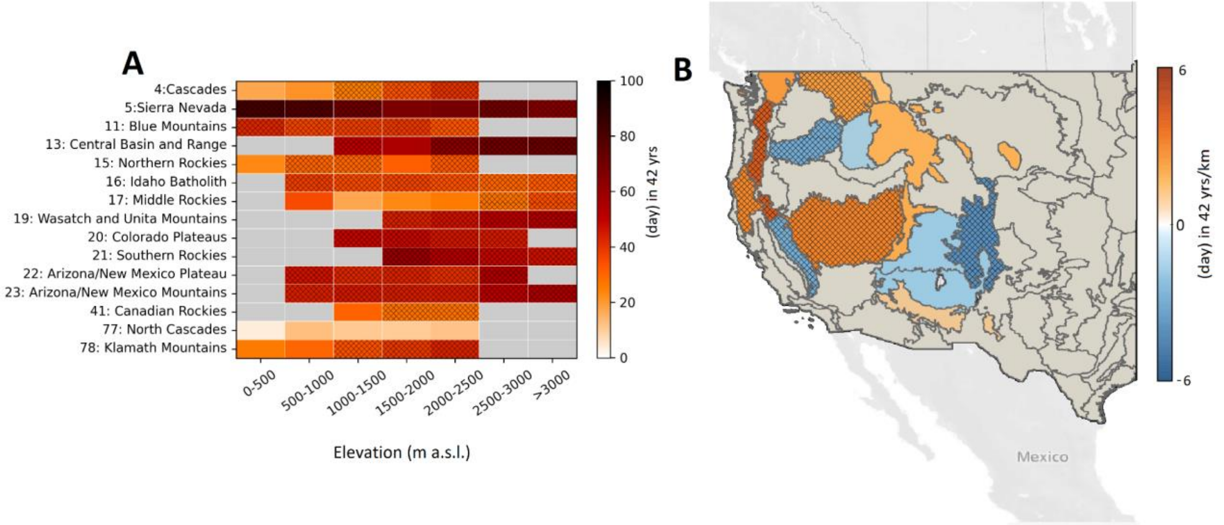

Figure S39. Elevation-dependent increase in extreme fire danger days. (A) *Temporal trends in extreme fire danger days – associated with daily ERC larger than the 95th percentile of long-term record from 1979-2020 – in each elevation band and ecoregion.* ***(B)*** *Slope of temporal trends in extreme fire danger days across elevation bands. Hatched areas indicate statistically significant trends at the 95% confidence level. (© OpenStreetMap contributors 2017. Distributed under the Open Data Commons Open Database License (ODbL) v1.0.)*[35].

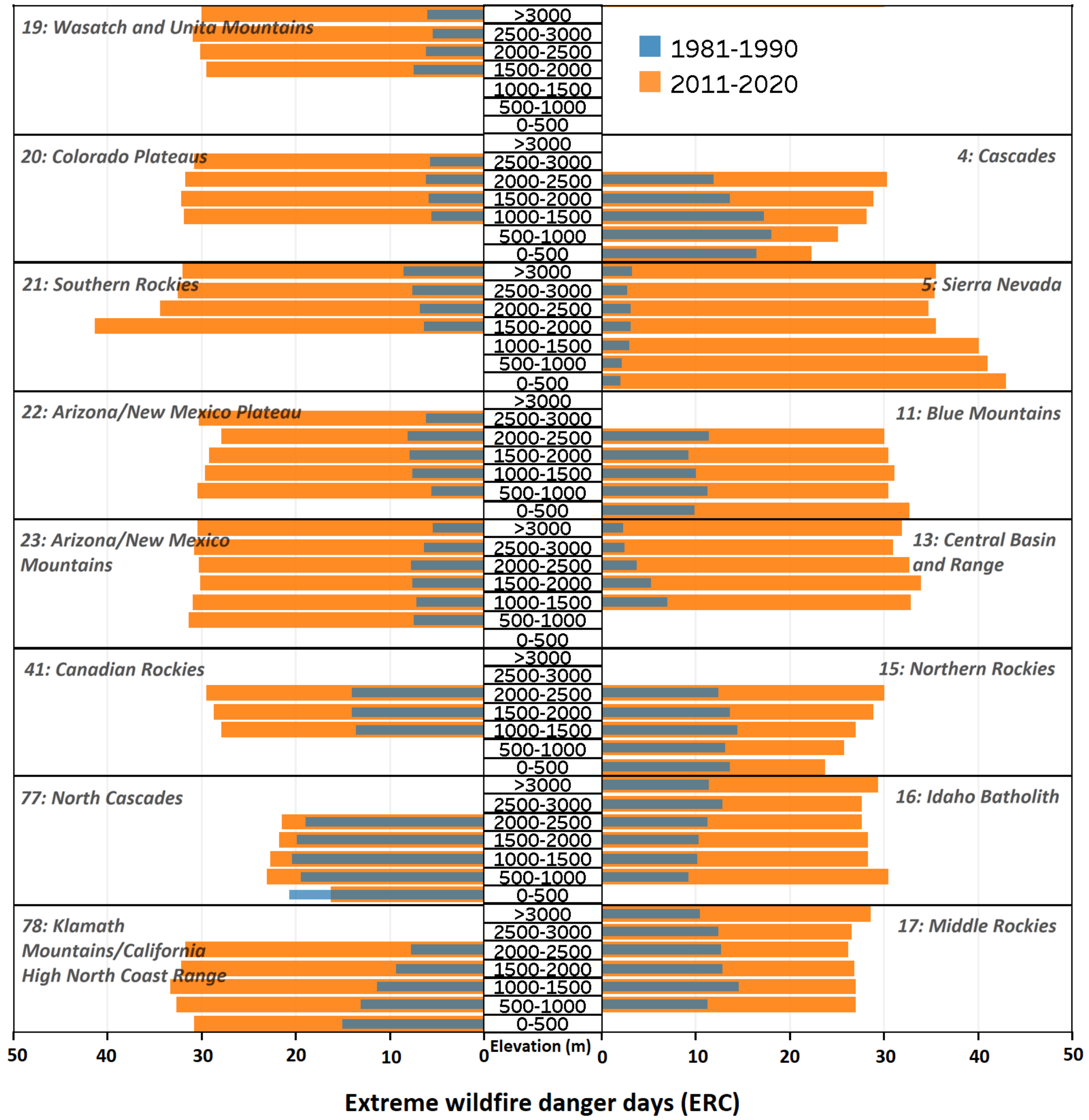

Figure S40. Annual extreme fire danger days associated with daily ERC larger than the 95th percentile of long-term record from 1979-2020. *Decadal average extreme fire danger days per year from 1981-1990 (blue) and 2011-2020 (orange).*

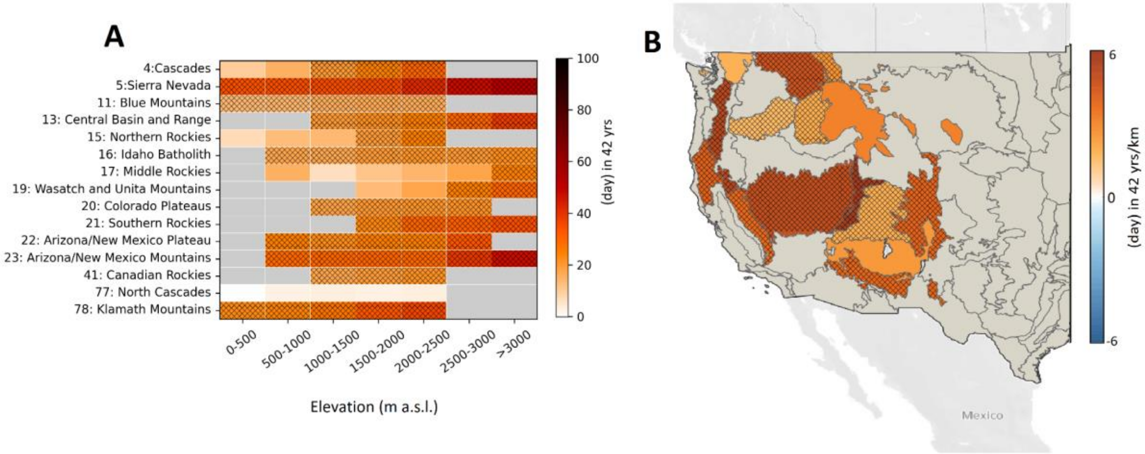

Figure S41. Elevation-dependent increase in high fire danger days. (A) *Temporal trends in high fire danger days – associated with daily VPD larger than the 75th percentile of long-term record from 1979-2020 – in each elevation band and ecoregion.* ***(B)*** *Slope of temporal trends in high fire danger days across elevation bands. Hatched areas indicate statistically significant trends at the 95% confidence level. (© OpenStreetMap contributors 2017. Distributed under the Open Data Commons Open Database License (ODbL) v1.0.)*[35]*.*

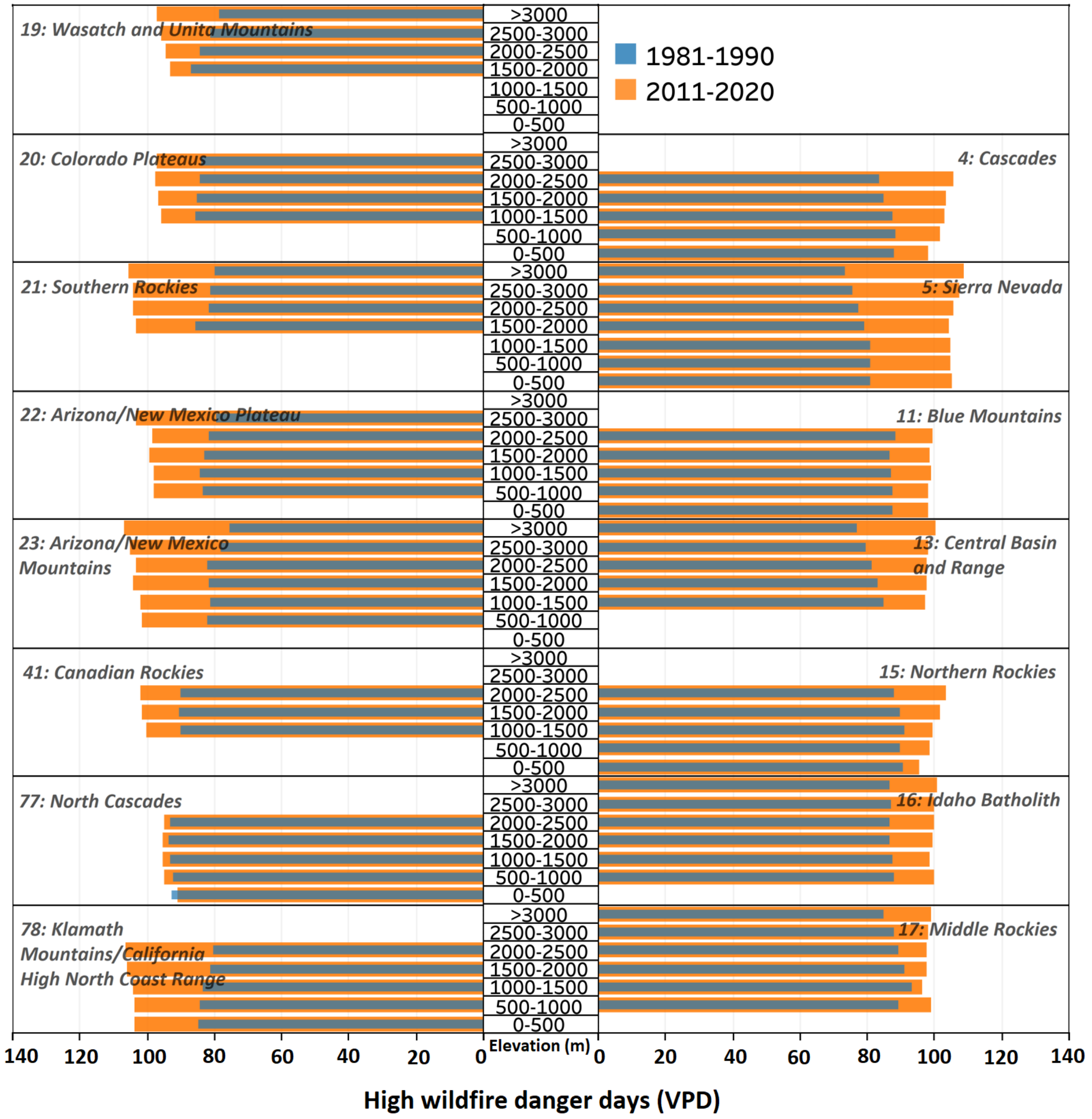

Figure S42. Annual high fire danger days associated with daily VPD larger than the 75th percentile of long-term record from 1979-2020. *Decadal average high fire danger days per year from 1981-1990 (blue) and 2011-2020 (orange).*

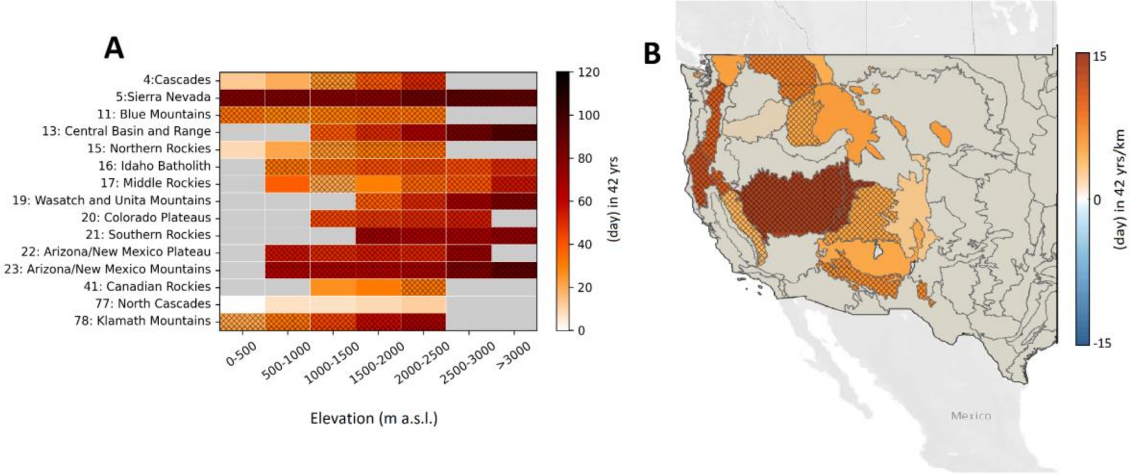

Figure S43. Elevation-dependent increase in high fire danger days. (A) *Temporal trends in high fire danger days – associated with daily FM100 smaller than the 25th percentile of long-term record from 1979-2020 – in each elevation band and ecoregion****. (B)*** *Slope of temporal trends in high fire danger days across elevation bands. Hatched areas indicate statistically significant trends at the 95% confidence level. (© OpenStreetMap contributors 2017. Distributed under the Open Data Commons Open Database License (ODbL) v1.0.)*[35]*.*

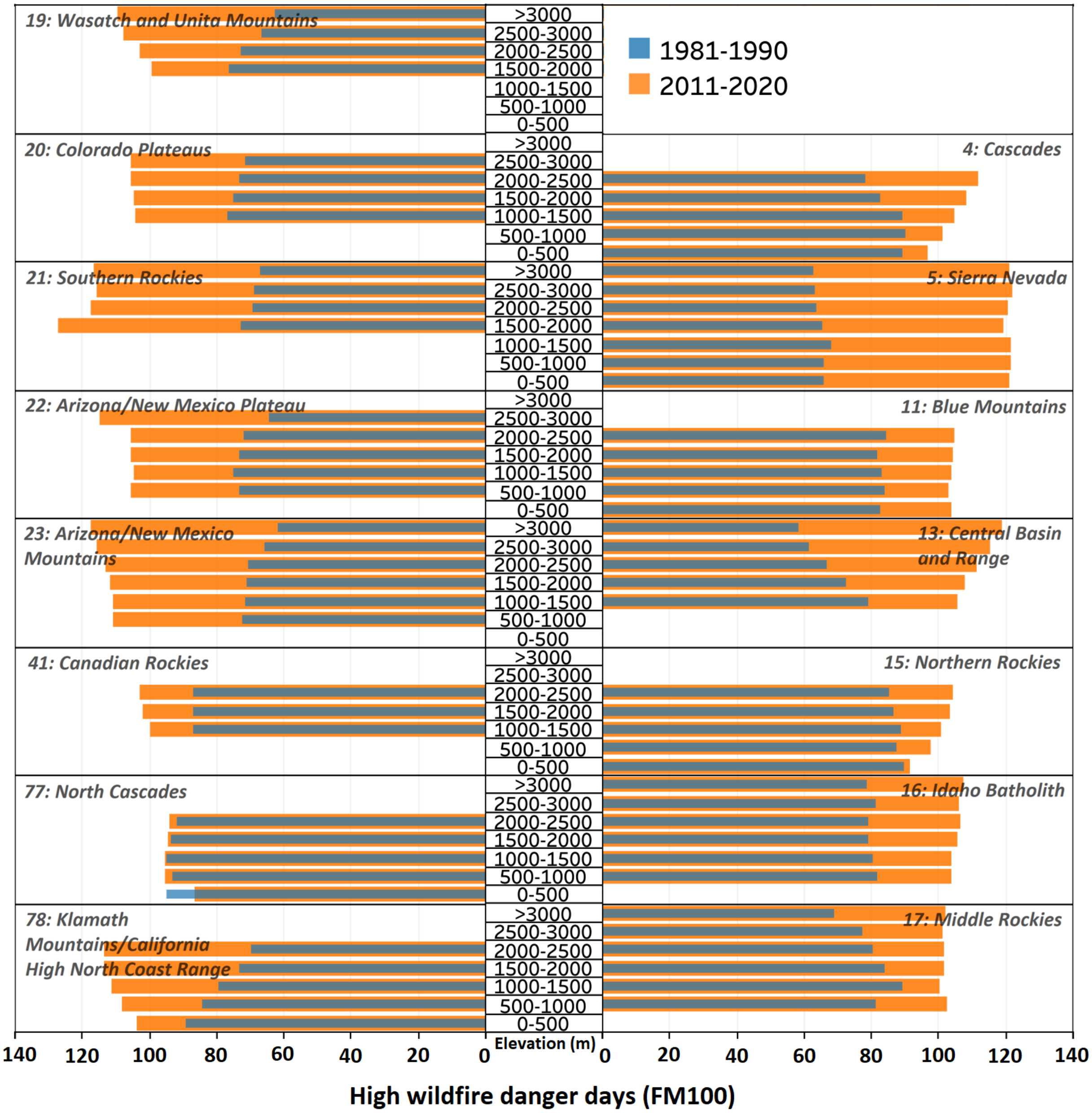

Figure S44. Annual high fire danger days associated with daily FM100 smaller than the 25th percentile of long-term record from 1979-2020. *Decadal average high fire danger days per year from 1981-1990 (blue) and 2011-2020 (orange).*

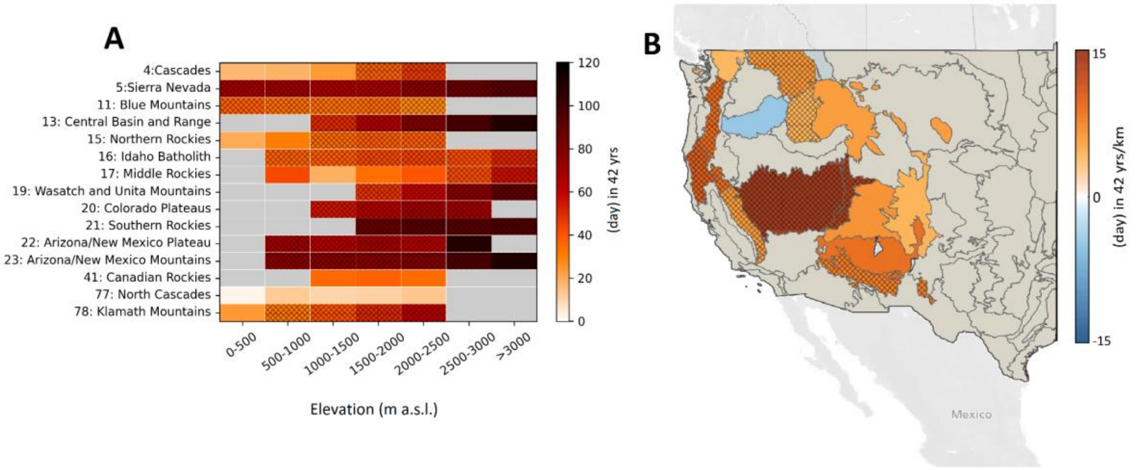

Figure S45. Elevation-dependent increase in high fire danger days. (A) *Temporal trends in high fire danger days – associated with daily FM1000 smaller than the 25th percentile of long-term record from 1979-2020 – in each elevation band and ecoregion.* ***(B)*** *Slope of temporal trends in high fire danger days across elevation bands. Hatched areas indicate statistically significant trends at the 95% confidence level. (© OpenStreetMap contributors 2017. Distributed under the Open Data Commons Open Database License (ODbL) v1.0.)*[35]*.*

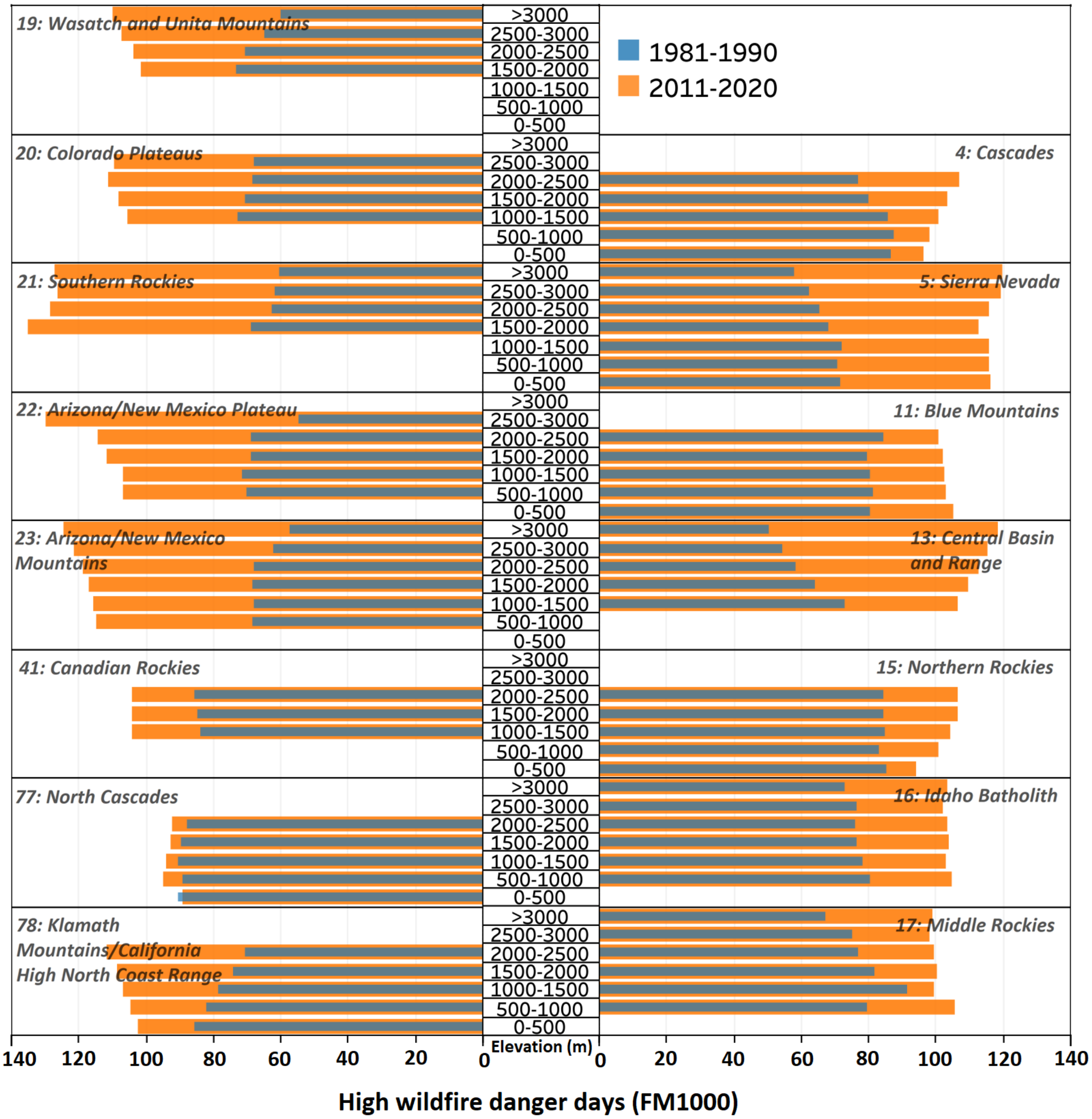

Figure S46. Annual high fire danger days associated with daily FM1000 smaller than the 25th percentile of long-term record from 1979-2020. *Decadal average high fire danger days per year from 1981-1990 (blue) and 2011-2020 (orange).*

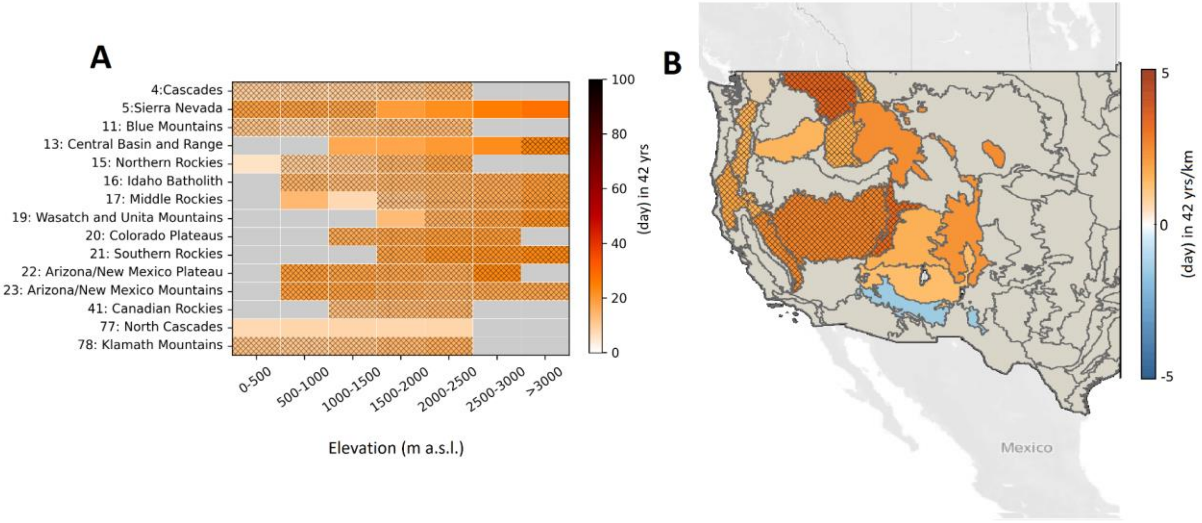

Figure S47. Elevation-dependent increase in extreme fire danger days. (A) *Temporal trends in extreme fire danger days – associated with daily VPD larger than the 95th percentile of long-term record from 1979-2020 – in each elevation band and ecoregion.* ***(B)*** *Slope of temporal trends in extreme fire danger days across elevation bands. Hatched areas indicate statistically significant trends at the 95% confidence level. (© OpenStreetMap contributors 2017. Distributed under the Open Data Commons Open Database License (ODbL) v1.0.)[35].*

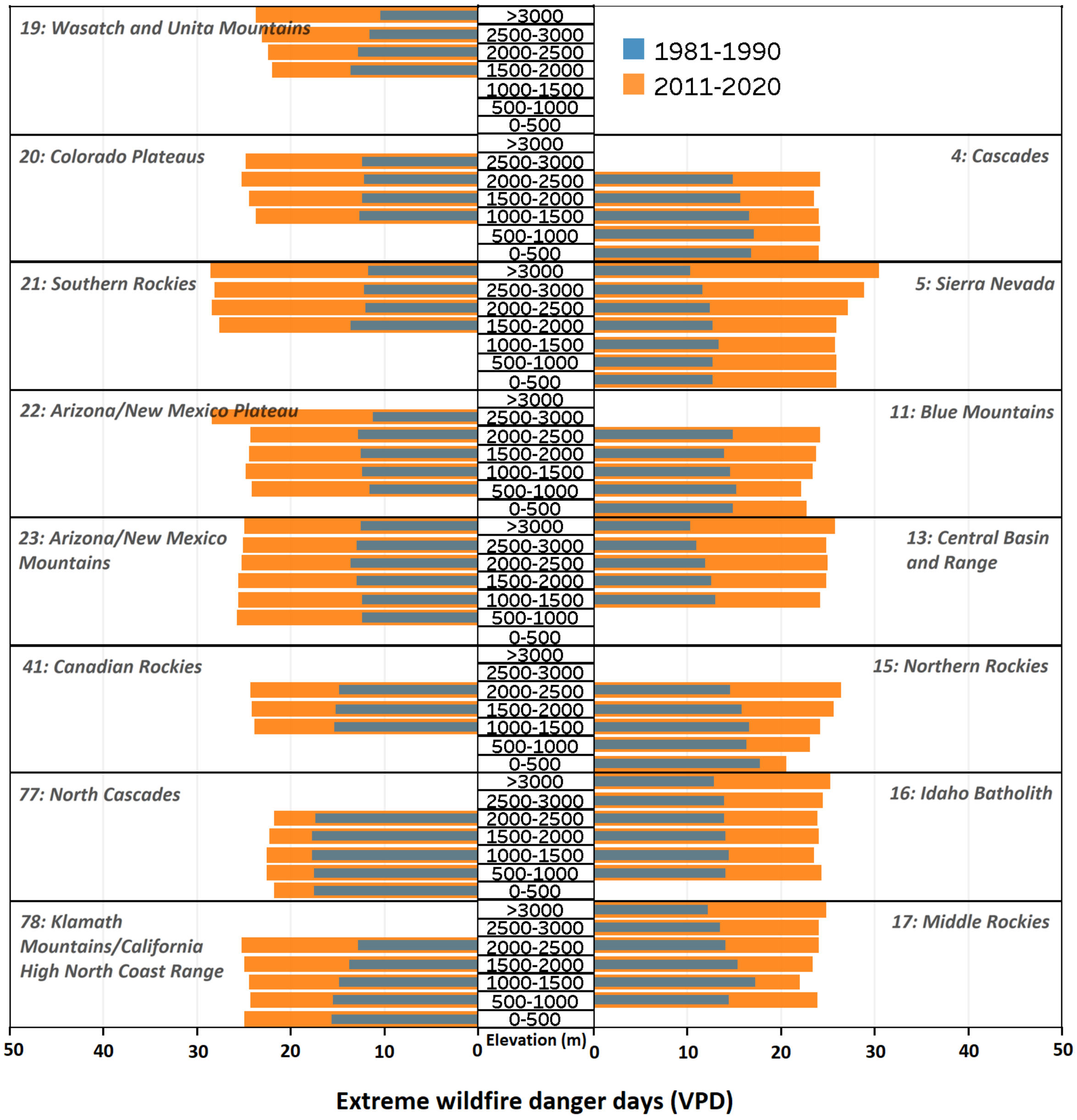

Figure S48. Annual extreme fire danger days associated with daily VPD larger than the 95th percentile of long-term record from 1979-2020. *Decadal average extreme fire danger days per year from 1981-1990 (blue) and 2011-2020 (orange).*

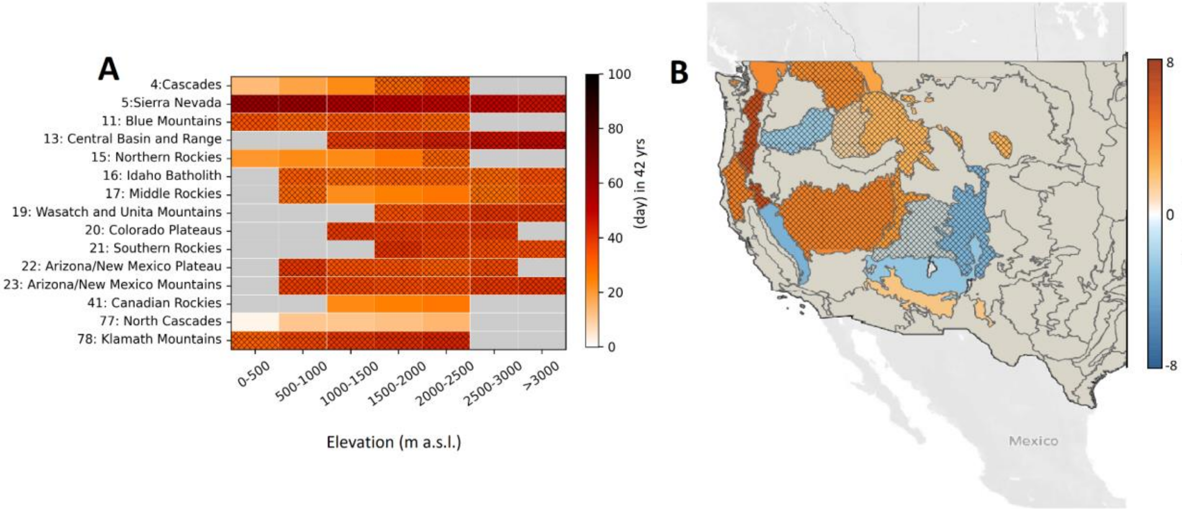

Figure S49. Elevation-dependent increase in extreme fire danger days. (A) *Temporal trends in extreme fire danger days – associated with daily FM100 smaller than the 5th percentile of long-term record from 1979-2020 – in each elevation band and ecoregion.* ***(B)*** *Slope of temporal trends in extreme fire danger days across elevation bands. Hatched areas indicate statistically significant trends at the 95% confidence level. (© OpenStreetMap contributors 2017. Distributed under the Open Data Commons Open Database License (ODbL) v1.0.)*[35]*.*

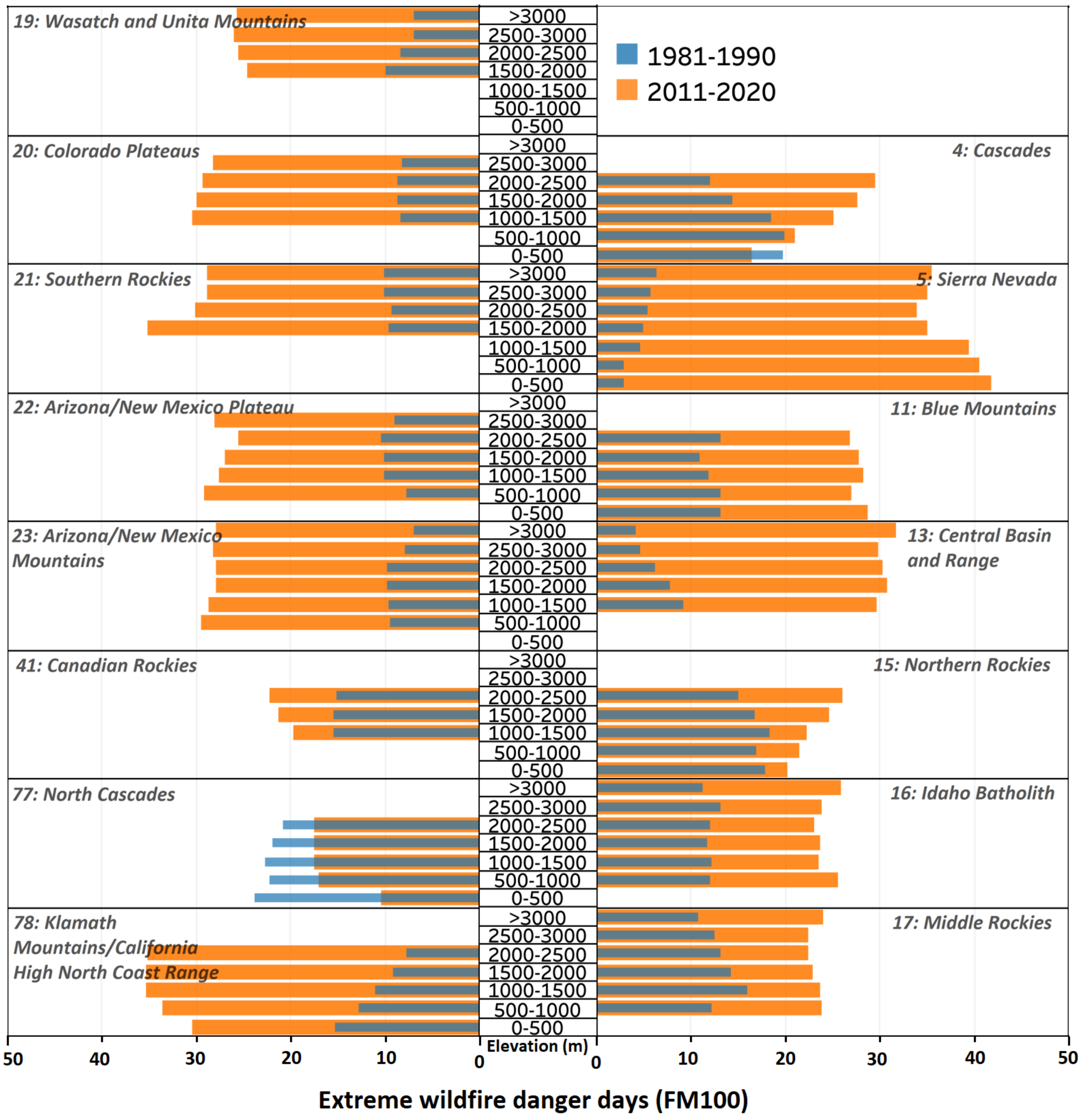

Figure S50. Annual extreme fire danger days associated with daily FM100 smaller than the 5th percentile of long-term record from 1979-2020. *Decadal average extreme fire danger days per year from 1981-1990 (blue) and 2011-2020 (orange).*

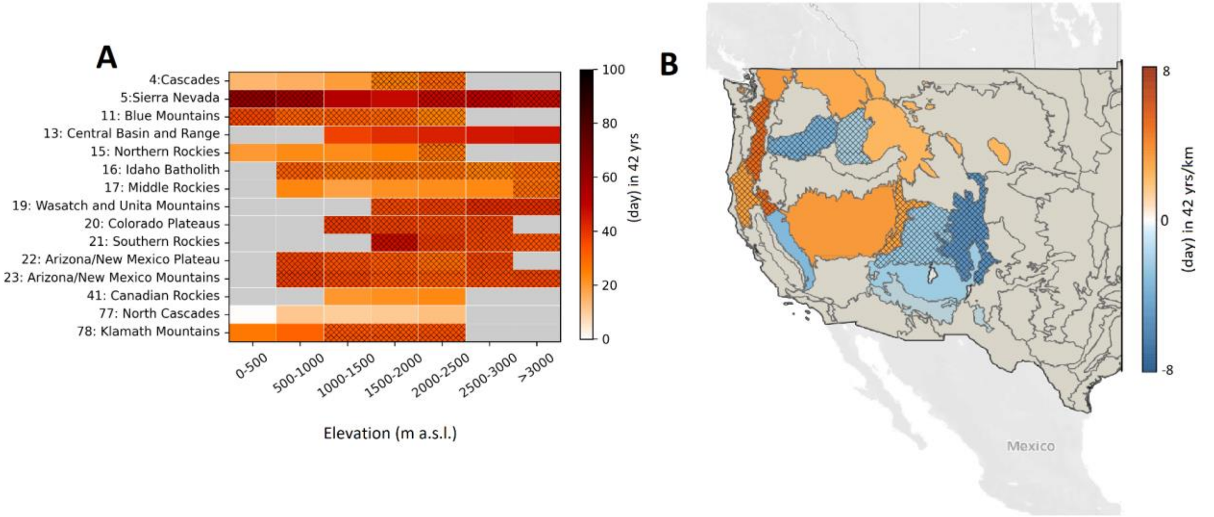

Figure S51. Elevation-dependent increase in extreme fire danger days. (A) *Temporal trends in extreme fire danger days – associated with daily FM1000 smaller than the 5th percentile of long-term record from 1979-2020 – in each elevation band and ecoregion.* ***(B)*** *Slope of temporal trends in extreme fire danger days across elevation bands. Hatched areas indicate statistically significant trends at the 95% confidence level. (© OpenStreetMap contributors 2017. Distributed under the Open Data Commons Open Database License (ODbL) v1.0.)*[35].

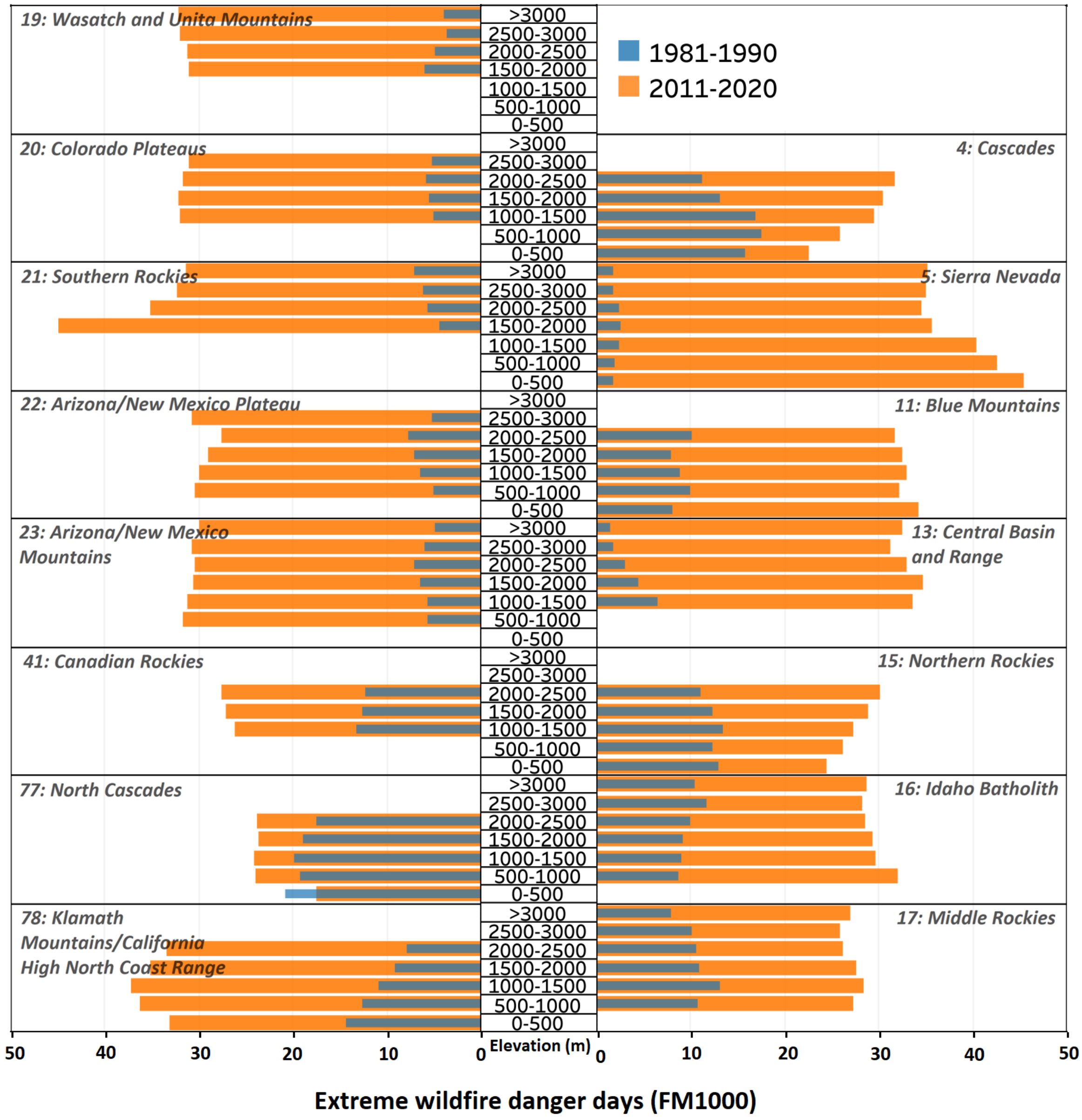

Figure S52. Annual extreme fire danger days associated with daily FM1000 smaller than the 5th percentile of long-term record from 1979-2020. *Decadal average extreme fire danger days per year from 1981-1990 (blue) and 2011-2020 (orange).*

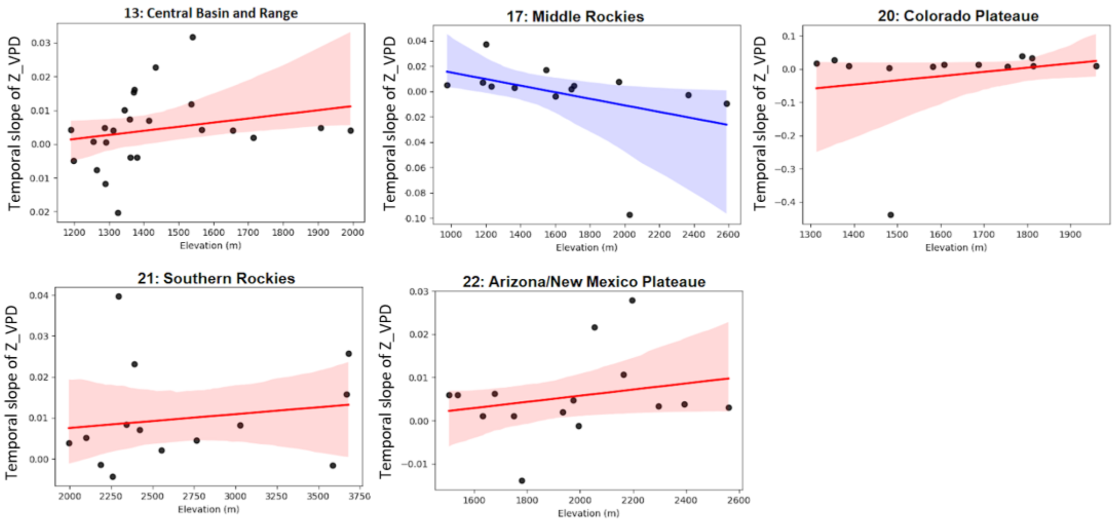

Figure S53. Elevational dependent fire danger intensification using ground observation data. *Trend line shows elevational gradient of temporal trends in z-score of average warm-season VPD (shown with a dot for each station). Positive values (red shades) indicate larger intensification of fire danger in higher elevations. We used vapor pressure deficit (VPD) as the fire danger metric, since VPD calculation only requires daily maximum and minimum temperature and relative humidity that are commonly observed in various weather stations – as the broader suite of observations needed to calculate ERC are resulting in a limited sample of stations. Since the response of VDP to temperature increase is exponential which inflates trends in lower elevation stations that have higher temperature baselines compared to the higher elevation stations, we used linear regression of temporal trends in the z-score of VPD in each station* $\left(z = \frac{x - mean(x)}{std(x)}\right)$*. Among available stations within the boundaries of mountainous ecoregions of the western US, we selected stations with at least ten years of continuous and consistent daily data (daily max and min temperature and relative humidity). We calculated daily VPD, and then averaged them over May through September to calculate average warm-season VPD. We then calculated z-score VPD for warm season averages. We subsequently calculated the linear regression trend slope of z-score VPD as a function of elevation for ecoregions that have at least 10 stations. Obviously, neither 10 years of data is sufficient for temporal trend calculation nor 10 stations across the elevation gradient is enough for robust elevational trend analysis, but given the scarcity of consistent data providing stations, we proceeded with this selection.*

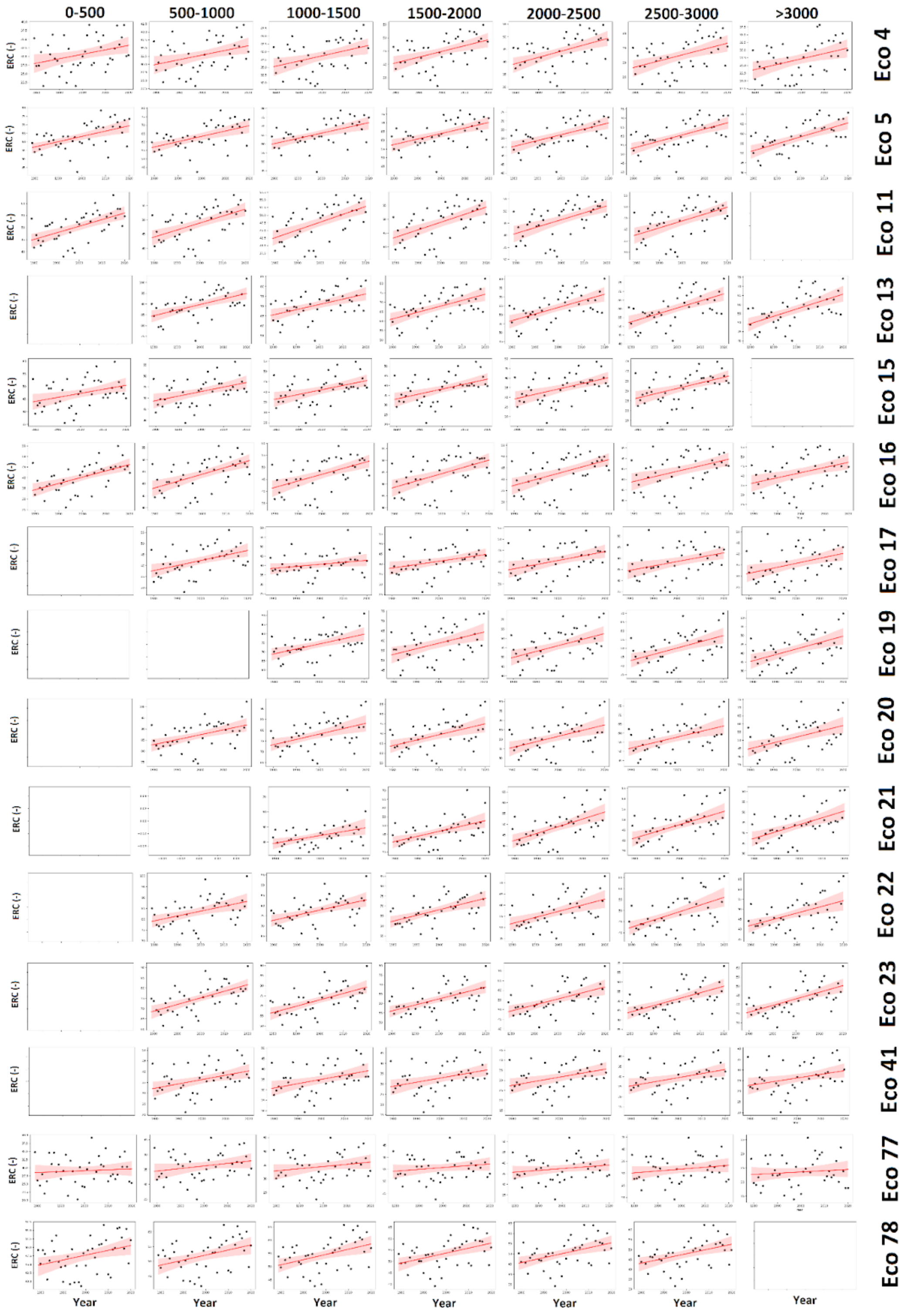

Figure S54. Annual warm-season ERC from 1979-2020 (blue dots), and temporal trends (shaded red line), in each elevation band in each ecoregion. *Elevation bands (with 500 m increments) are presented as columns and each ecoregion is presented in one row. For ecoregion names refer to* ***Table S1****.*
