# Supplementary material for: Elevation-dependent intensification of fire danger in the western United States
Source: Nat Commun. 2023 Mar 30;14:1773. doi: 10.1038/s41467-023-37311-4 (PMC10063545; doi:10.1038/s41467-023-37311-4)
Supplement: Supplementary file 3 — Description of Additional Supplementary Files [file 41467_2023_37311_MOESM3_ESM.pdf]

Description of Additional Supplementary Files

File Name: Supplementary Data 1
Description: May-September average of fire danger indices and meteorological variables for each elevation band and each ecoregion from 1979-2020.
